# Gut Microbiota‐Dependent Tryptophan Metabolism Drives the Therapeutic Effects of Baitouweng Tang in Colitis via the AhR Pathway

**DOI:** 10.1002/fsn3.72434

**Published:** 2026-10-14

**Authors:** Chun Niu, Yongli Hua, Jianguo Kang, Fang Li, Baoxia Chen, Rongxia Guo, Yanming Wei

**Affiliations:** ^1^ Institute of Traditional Chinese Veterinary Medicine, College of Veterinary Medicine Gansu Agricultural University Lanzhou China; ^2^ Huating Animal Husbandry and Veterinary Bureau Huating China

**Keywords:** AhR, Baitouweng Tang, colitis, gut microbiota, indole‐3‐lactic acid

## Abstract

With dysregulation of the gut microbiota and its metabolic byproducts playing a pivotal role in inflammatory bowel disease (IBD) pathogenesis. Baitouweng Tang (BTWT), a traditional herbal formulation, has long been employed to treat gastrointestinal disorders and shows promising potential in the management of IBD. However, its precise pharmacological mechanisms remain elusive. In this study, we employed targeted metabolomics, high‐throughput sequencing, and molecular biology techniques to elucidate the therapeutic mechanisms of BTWT in the treatment of ulcerative colitis (UC). BTWT significantly enhanced the relative abundance of *Bifidobacterium*, improved the indole metabolic pathway of tryptophan, and restored the levels of indole metabolites, such as indole‐3‐lactic acid (ILA). These changes activated the aryl hydrocarbon receptor (AhR) and led to marked improvements in the clinical symptoms of UC in mice. Conversely, the therapeutic effects of BTWT were substantially diminished in an antibiotic‐induced gut microbiota depletion model. Notably, fecal microbiota transplantation (FMT) partially restored BTWT efficacy, underscoring the critical role of the gut microbiota in mediating its therapeutic effects. Furthermore, in an in vitro lipoteichoic acid (LTA)‐induced HT‐29 inflammation model, ILA treatment attenuated inflammation and concurrently increased the expression of AhR, CYP1A1, and CYP1B1. These findings suggest a potential link between BTWT‐induced changes in tryptophan metabolites, AhR pathway activation, and the amelioration of colitis, though the causal relationship remains to be established. Our study provides a basis for further mechanistic investigation into the AhR pathway as a possible therapeutic target in UC.

## Graphical abstract

Baitouweng Tang alleviates colitis symptoms in mice by regulating the structure and diversity of gut microbiota, increasing the content of gut microbiota‐related tryptophan metabolites, activating AhR, inhibiting the release of inflammatory mediators, and improving intestinal homeostasis.

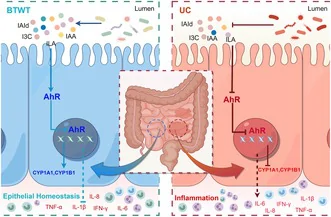

## 1 Introduction

Ulcerative colitis (UC) is a leading form of inflammatory bowel disease (IBD) (Kobayashi et al. 2020). The pathogenesis of UC is multifactorial and complex and is believed to involve factors such as intestinal dysbiosis, dysfunction of the intestinal epithelial barrier, impaired immune response, and genetic predisposition. These factors are closely associated with inflammation, which is characterized by excessive activity, clinical remission, and a tendency for relapse. The World Health Organization (WHO) categorizes ulcerative colitis as a contemporary, treatment‐resistant condition (Rosen et al. 2015; Ungaro et al. 2017; Yoshida et al. 2015). UC begins with rectal mucosal membrane inflammation, extends proximally, accumulates in the colonic mucosa and submucosa, and is a persistent diffuse inflammation with typical clinical symptoms of abdominal pain, diarrhea, and bloody stools (Feuerstein et al. 2019). Currently, drugs commonly used to treat UC are 5‐aminosalicylic acid, cortisol, glucocorticoids, immunosuppressants, etc. However, prolonged use of these drugs frequently leads to a range of adverse reactions and side effects (Grossberg et al. 2022; Guo et al. 2022; Li, Wang, Ma, et al. 2022; Magro et al. 2020; Niu et al. 2021). Therefore, the search for alternative drugs with fewer adverse reactions and side effects is a promising strategy, which is of great significance in treating UC. Chinese herbal medicines are derived primarily from plants. It has many targets, definite curative effects, low recurrence rates, and fewer toxic side effects. Its unique advantages are evident in UC (Liu et al. 2022; Shen et al. 2019).

Baitouweng Tang (BTWT) is a formula from Zhang Zhongjing's Shanghai Lun, which consists of four herbs *Pulsatilla chinensis* (Bunge) Regel (Bai Tou Weng), *Phellodendron chinense* C. K. Schneid. (Huang Bai), and *Coptis chinensis* Franch. [Ranunculaceae] (Huang Lian) and *Fraxinus chinensis* Roxb. [Oleaceae] (Qin Pi) is known for its ability to cool the blood and stop diarrhea. BTWT is used as the main treatment for febrile dysentery, anal burning, and dysentery with pus and blood and commonly applied in medical settings to treat various gastrointestinal disorders, which is a typical representative formula for the treatment of UC (Liu et al. 2020; Niu et al. 2023). Despite this, the precise pharmacodynamic pathways remain incompletely characterized. Extensive research has revealed that the gut microbiome is a key player in both the onset and advancement of ulcerative colitis, with microbial imbalances frequently triggering this inflammatory condition (Ni et al. 2017; Yu 2018). Small‐molecule metabolites produced by gut flora metabolism, including bile acids, short‐chain fatty acids, and tryptophan metabolites, are intermediates in coordinating microbial‐host interactions and play a vital role in preserving gut immunity and epithelial barrier integrity (Lavelle and Sokol 2020). Alterations in the levels of these small molecules produced by gut flora metabolism are associated with the occurrence of UC (Lloyd‐Price et al. 2019). The modified tryptophan metabolic changes seen in IBD could significantly contribute to the disease's pathogenesis (Agus et al. 2018). Many small molecules produced by the metabolism of tryptophan by the gut microbiota are endogenous ligands for the aryl hydrocarbon receptor (AhR), a signaling molecule for the activation of AhR. The metabolic levels of these small molecules are altered in UC changed (Gasaly et al. 2021; Yang and Cong 2021). Normally, AhR remains dormant in the cytoplasm, where it forms a complex with HSP90, XAP2 (X‐Associated Protein‐2), and p23, the chaperone protein of HSP90. Upon binding to its ligand, the AhR‐HSP90 complex is activated, leading to a conformational change that results in the release of HSP90. This activation causes the AhR receptor to translocate to the nucleus, where it interacts with the xenobiotic response element and initiates the transcriptional regulation of downstream target genes (Li, Wang, Su, et al. 2022). AhR activation induces the expression of downstream genes such as interleukin‐22 (IL‐22), thereby maintaining intestinal homeostasis (Sun et al. 2020). Recent studies have found that BTWT promotes tryptophan metabolism by promoting DOPA decarboxylase and alleviates ulcerative colitis (Zhang, Lin, et al. 2024). In addition, BTWT promotes the enrichment of Trp metabolite IPA derived from microflora, inhibits RIPK1/RIPK3/MLKL signaling pathway, inhibits the necrosis of intestinal epithelial cells and improves barrier function to alleviate colitis (Hu et al. 2025). Therefore, we speculate that BTWT may alleviate colitis by regulating intestinal microbiota and related tryptophan metabolism to activate AhR.

## 2 Reagents and Methods

### 2.1 Preparation of BTWT

Bai Tou Weng, Huang Bai, Huang Lian, and Qin Pi were purchased from the Yellow River Herb Market in Lanzhou, Gansu. Bai Tou Weng, Huang Bai, Huang Lian, and Qin Pi were weighed accurately according to the ratio of 4:3:2:4 and extracted by refluxing with distilled water three times, each time for 1 h. The amount of water added was 10, 8, and 8 times the amount of raw herbs, filtered, combined with the filtrate obtained from the three extractions, decompression concentration, partially stored at 4°C, and partially freeze‐dried.

### 2.2 BTWT Composition Analysis

Approximately 100 mg of the sample was weighed, 1 mL of aqueous solution (with mixed internal standard, 4 μg/mL L‐2‐chlorophenylmalonic acid) was added, vortexed, and shaken, and then steel beads were added, and then pre‐cooled in the refrigerator at −40°C for 2 min, and then put into the grinder for grinding (60 HZ, 2 min); the extraction was performed by ultrasound in an ice‐water bath for 60 min. The sample was extracted by ultrasonication (60 HZ, 2 min) in an ice‐water bath for 60 min, and then left at −40°C for 30 min. The sample was centrifuged at 12,000 rpm for 10 min at 4°C and diluted 10‐fold with methanol (containing 4 μg/mL L‐2‐chlorobenzene propanoic acid as the internal standard). Then, 200 μL of the supernatant was transferred into a tube‐lined LC–MS injection vial for analysis.

### 2.3 Experimental Animal

Male C57BL/6 mice were purchased from the Laboratory Animal Center, Lanzhou Institute of Veterinary Medicine, Chinese Academy of Agricultural Sciences under license No. SCXK (Gan) 2022–0002. The mice were provided with ad libitum water and standard chow, and were housed at a temperature of 22°C ± 1°C, humidity of 50% ± 5%, and a 12‐h light/12‐h dark cycle. The animal experiments were approved by the Animal Ethics Committee of Gansu Agricultural University and the Institutional Animal Care and Use Committee (License Approval No.: GSAU‐Eth‐VMC‐2023‐041), and the welfare of the experimental animals was ensured to the maximum extent possible during the experiments.

### 2.4 Establishment of a Pseudo‐Germ‐Free Mouse Model

C57BL/6 mice were acclimatized for 1 week, after which they were randomly assigned to a control group (K) or a mixed antibiotic‐treated model group (ABX), with 10 mice in each group. The ABX group was gavaged once a day in the morning and once in the evening with a mixture of antibiotics (Vancomycin 0.05 g/kg, Ampicillin 0.1 g/kg, Metronidazole 0.1 g/kg, and Neomycin sulfate 0.1 g/kg, purchased from Shanghai McLean Ltd.) for 10 consecutive days. These methods have been reported in previous studies and have been modified according to the actual situation (Zarrinpar et al. 2018). Mice were euthanized on day 11, and cecal contents were collected for 16SrRNA sequencing analysis.

### 2.5 Establishment of a Mouse Model of Colitis and Intervention With BTWT

At the end of the acclimatization period, the mice were randomly assigned to the control (CON), colitis (UC), Baitouweng Tang high dose (BTWT‐H), Baitouweng Tang medium dose (BTWT‐M), Baitouweng Tang low dose (BTWT‐L), and salazosulfapyridine (SASP) positive groups, with ten mice in each group. The control group received distilled water ad libitum daily, while mice in all other groups were given 2.5% DSS ad libitum for seven consecutive days to establish an acute UC model. CON and UC were intervened by intragastric administration of distilled water every day, and BTWT high, medium, and low dose groups were intervened by intragastric administration of 8.1 g/kg, 5.4 g/kg, and 2.7 g/kg BTWT every day.

For the UC model after antibiotic depletion, on the basis of the established microbial depletion model mice, the colitis model (ABX‐UC) was established by giving 2.5% DSS for seven consecutive days; on this model, Baitouweng Tang intervention (ABX‐BTWT) was given, with ten mice in each group. After the experiment, the required samples were collected for subsequent analysis.

### 2.6 Fecal Microbiota Transplantation (FMT)

C57BL/6 mice were divided into the control (Control), UC transplantation (FMT‐UC), and BTWT transplantation (FMT‐BTWT) groups. Before FMT transplantation, recipient mice were used to deplete intestinal microorganisms according to the method described above (intragastric administration of mixed antibiotics). Fresh feces from donor mice were collected daily, dissolved in saline at a 1:10 (w/v) ratio, vortexed for 1 min, and then centrifuged at 500 ***g*** for 3 min to remove particulate matter. The resulting supernatant was gavaged (200 μL/mouse) within 10 min, twice daily.

### 2.7 Disease Activity Index (DAI)

DAI calculated and statistically analyzed daily during the animal experiments in reference (Xiao et al. 2024).

### 2.8 Hematoxylin–Eosin Staining

Mouse colon tissues fixed in a 10% formalin solution were routinely dehydrated, embedded, sectioned, stained according to the procedure, and scored histopathologically according to the criteria (Xiao et al. 2024).

### 2.9 Alisin Blue‐Periodic Acid Schiff Staining

Mouse colon tissue sections were stained using the Alisin Blue‐Periodic Acid Schiff Staining Kit and counted for changes in goblet cells.

### 2.10 16S rRNA Analysis

Genomic DNA from the microbial community was extracted according to the protocol provided with the E.Z.N.A. Soil DNA Kit (Omega Bio‐tek, Norcross, GA, USA). The extracted DNA was used as a template for PCR amplification of the V3‐V4 variable region of the 16S rRNA gene, using the forward primer 338 F (5′‐ACTCCTACGGGAGGGAGGCAGCAG‐3′) and the reverse primer 806 R (5′‐GGACTACHVGGGTWTCTAAT‐3′). High‐throughput 16S rRNA sequencing was performed by Majorbio Bio‐Pharm Technology Co. Ltd., and the data were analyzed using the I‐Sanger Cloud Platform, a free online resource.

### 2.11 Targeted Tryptophan Metabolism Detection

An appropriate amount of the standard substance of the tryptophan metabolic pathway was accurately weighed and a single standard mother liquor was prepared using 50% methanol. An appropriate amount of isotope standard (Trp‐D5) was accurately weighed, a single standard mother liquor was prepared with methanol, and an isotope internal standard working solution with a concentration of 4000 ng/mL was diluted with ultrapure water. Separation was performed using a Nexera Series LC‐40 liquid chromatograph equipped with an ACQUITY UPLC HSS T3 column (2.1 × 150 mm, 1.8 μm, Waters Corporation, USA) at 40°C, with an injection volume of 2 μL. The mobile phase consisted of 0.1% formic acid in water (Phase A) and 0.1% formic acid in acetonitrile (Phase B). The chromatographic gradients (including time, flow rate, A% and B%) are shown in Table 1. Mass spectrometry was performed using an AB SCIEX QTRAP 6500+ in both positive and negative mode. The conditions were as follows: Curtain Gas (CUR) at 35, Collision Gas (CAD) set to medium, IonSpray Voltage (IS) at 5500/−4500, Temperature (TEM) at 550°C, Ion Source Gas 1 (GS1) at 50, and Ion Source Gas 2 (GS2) at 50. A linear regression standard curve was generated using the ratio of the analyte's peak area to that of the internal standard as the vertical axis, and the analyte concentration as the horizontal axis. The method validation results are shown in Tables 2 and 3. The calibration curves were linear over the tested ranges (*R* ^2^ > 0.99), and the recoveries met the acceptable criteria (85%–115%) as detailed in Tables 2 and 3.

**TABLE 1:** Chromatographic gradient.

Time (min)	Flow rate (mL/min)	A%	B%
0.00	0.35	99	1
2.5	0.35	89	11
5.5	0.35	89	11
6.5	0.35	72	28
7.5	0.35	72	28
12.5	0.35	50	50
13.5	0.35	5	95
15.5	0.35	5	95
15.6	0.35	99	1
18	0.35	99	1

**TABLE 2:** Standard curve and limit of quantitation of tryptophan pathway substance line.

Analyte	Linear range (ng/mL)	Linear equation	Correlation coefficient *R* ^2^	Quantitative limit (ng/mL)
Indole	17.92–20,000	*y* = 0.01161*x* + −0.00129	0.99307	17.9200
Indole‐3‐lactic Acid	0.8960–1000	*y* = 0.97968*x* + 0.00865	0.99293	0.8960
5‐Hydroxyindole‐3‐acetic acid	0.4480–500	*y* = 3.04144*x* + 0.01194	0.99457	0.4480
Indole‐3‐acetic acid	0.1344–150	*y* = 2.61028*x* + 0.00131	0.99424	0.1344
5‐hydroxytryptophan	0.4480–500	*y* = 2.77729*x* + 0.00296	0.99716	0.4480
3‐Hydroxy‐DL‐kynurenine	0.4480–500	*y* = 1.83383*x* + 0.00366	0.99471	0.4480
Nicotinamide (Vitamin B3)	0.1120–125	*y* = 5.13577*x* + 0.00219	0.99342	0.1120
3‐Indoleacetonitrile	0.4480–500	*y* = 1.03175*x* + 0.00221	0.99663	0.4480
Nicotinic Acid	0.1792–200	*y* = 2.57477*x* + 0.00570	0.99607	0.1792
3‐hydroxyanthranilic acid	0.4480–500	*y* = 3.22158*x* + 0.00293	0.99754	0.4480
N‐Acetyl‐serotonin	0.0358–40	*y* = 34.20802*x* + 0.00858	0.99136	0.0358
L‐Tryptophan	2.24–2500	*y* = 1.23681*x* + 0.01203	0.99329	2.2400
Kynurenine	1.3440–1500	*y* = 2.3747*x* + 8.78688e‐4	0.99805	1.3440
Kynurenic Acid	0.2240–250	*y* = 14.54267*x* + 0.01499	0.99452	0.2240
Tryptamine	0.0448–50	*y* = 5.01523*x* + 0.00272	0.99755	0.4480
Serotonin	0.7168–800	*y* = 2.09289*x* + 0.00158	0.99447	0.7168
Tryptophol	0.4480–500	*y* = 0.65267*x* + −0.00315	0.99254	0.4480
Xanthurenic acid	0.1120–125	*y* = 9.90233*x* + 0.00364	0.99845	0.1120

**TABLE 3:** Precision and recovery of tryptophan pathway substances.

Analyte	Concentration (ng/mL)	Intra‐day precision (%) (*n* = 6)	Inter‐day precision (%) (*n* = 18)	Recovery (%) (*n* = 6)
Indole	51.2	4.53	5.21	104.26 ± 4.72
	10,000	3.44	4.51	100.22 ± 3.45
	15,000	2.29	2.62	103.78 ± 2.37
Indole‐3‐lactic Acid	2.56	1.53	5.80	109.38 ± 1.67
	500	7.86	5.14	97.93 ± 7.69
	750	3.55	3.38	96.01 ± 3.41
5‐Hydroxyindole‐3‐acetic acid	1.28	7.31	6.35	102.65 ± 7.50
	250	2.16	4.10	98.75 ± 2.14
	375	1.67	3.29	99.07 ± 1.66
Indole‐3‐acetic acid	0.384	5.30	9.49	107.86 ± 5.72
	75	7.08	5.70	97.88 ± 6.93
	112.5	1.65	6.11	101.36 ± 1.67
5‐hydroxytryptophan	1.28	8.94	6.30	102.28 ± 9.14
	250	3.93	2.82	101.7 ± 4.00
	375	2.08	3.46	100.94 ± 2.10
3‐Hydroxy‐DL‐kynurenine	1.28	4.41	6.50	103.96 ± 4.58
	250	3.70	2.89	101.03 ± 3.74
	375	3.93	4.05	103.11 ± 4.05
Nicotinamide (Vitamin B3)	0.32	1.94	5.44	110.66 ± 2.15
	62.5	3.11	4.00	98.15 ± 3.05
	93.75	2.70	2.84	96.7 ± 2.61
3‐Indoleacetonitrile	1.28	8.73	6.55	104.27 ± 9.11
	250	2.19	2.16	104.29 ± 2.29
	375	2.73	4.62	108.11 ± 2.95
Nicotinic Acid	0.512	4.99	6.08	108.84 ± 5.43
	100	2.93	4.26	93.37 ± 2.74
	150	1.26	2.55	93.4 ± 1.18
3‐hydroxyanthranilic acid	1.28	8.48	7.55	96.73 ± 8.21
	250	2.73	3.00	105.01 ± 2.87
	375	1.34	3.27	107.47 ± 1.44
N‐Acetyl‐serotonin	0.1024	4.02	4.87	106.69 ± 4.29
	20	3.03	3.45	100.21 ± 3.03
	30	4.25	3.10	94.25 ± 4.00
L‐Tryptophan	6.4	3.29	4.45	107.01 ± 3.52
	1250	4.43	3.17	99.13 ± 4.39
	1875	2.02	1.93	97.53 ± 1.97
Kynurenine	3.84	5.02	8.89	109.97 ± 5.52
	750	2.91	3.17	103.19 ± 3.00
	1125	2.41	3.36	104.01 ± 2.50
Kynurenic Acid	0.64	2.44	4.41	108.74 ± 2.65
	125	3.29	3.77	101.98 ± 3.35
	187.5	1.09	2.22	96.44 ± 1.05
Tryptamine	0.128	9.85	7.83	99.82 ± 9.84
	25	4.98	4.95	101.54 ± 5.05
	37.5	5.28	4.74	98.8 ± 5.21
Serotonin	2.048	4.68	8.02	110.71 ± 5.18
	400	6.47	5.66	101.39 ± 6.57
	600	6.64	4.76	98.14 ± 6.52
Tryptophol	1.28	2.39	8.69	110.85 ± 2.65
	250	4.05	3.52	99.48 ± 4.03
	375	3.55	3.83	94.2 ± 3.34
Xanthurenic acid	0.32	6.54	8.46	107.03 ± 6.99
	62.5	2.44	4.43	102.36 ± 2.50
	93.75	2.96	3.06	103.9 ± 3.08

### 2.12 Cell Culture

HT‐29 cells were purchased from Pricella Biotechnology Co. Ltd. and cultured in McCoy's 5A medium containing 10% fetal bovine serum (FBS), 1% penicillin–streptomycin, and 89% medium at 37°C with 5% CO_2_. After resuscitating the cells, they were continuously passaged thrice and used in the experiment. To model inflammation, the cells reached 80% confluence, were digested with trypsin, and cultured in 6‐well plates. After 24 h, the cells were stimulated with 5 μg/mL LTA for 6 h, followed by treatment with ILA for 12 h. Total protein and RNA were then extracted for analysis.

### 2.13 CCK8 Assay

When HT‐29 cells reached the logarithmic growth phase, trypsin digestion was used to prepare cell suspensions, inoculated in a 96‐well plate at 1 × 10^5^ cells/mL, 100 μL per well, and cultured in adherence to the wall for 24 h, then different concentrations of BTWT, ILA, and LTA were cultured for 6 h, 12 h or 24 h, respectively, and the viability of cells was detected by CCK‐8 reagent.

### 2.14 Quantitative Real‐Time Polymerase Chain Reaction

Total RNA from mouse colon tissue and cells was extracted using TransZol. The extracted RNA was then reverse‐transcribed into cDNA, which was subsequently amplified and detected by SYBR Green real‐time polymerase chain reaction. The target gene levels were normalized to GAPDH gene levels, and the $2^{-∆∆C_{t}}$ method was used to calculate the relative expression of the target genes. The primer sequence is as follows: mouse *Ahr*: 5′‐ACATCACCTATGCCAGCCG‐3′ and 5′‐GACTTAATTCCTTCAGCGGGGA‐3′; mouse *Cyp1a1*: 5′‐GGGTTTGACACAGTCACAACT‐3′ and 5′‐GGGACGAAGGATGAATGCCG‐3′; mouse Cyp1b1: 5′‐CACCAGCCTTAGTGCAGACAG‐3′ and 5′‐GAGGACCACGGTTTCCGTTG‐3′; mouse *Ifn‐γ*: 5′‐CAAGACTGTGATTGCGGGGT‐3′ and 5′‐GACATCTCCTCCCATCAGCA‐3′; mouse *Il‐6*: 5′‐TTGCCTTCTTGGGACTGATG‐3′ and 5′‐TCATTTCCACGATTTCCCAG‐3′; mouse *Il‐22*: 5′‐CTTGTGCGATCTCTGATGGCT‐3′ and 5′‐CCAGCATAAAGGTGCGGTTG‐3′; mouse *Gapdh*: 5′‐GGTTGTCTCCTGCGACTTCA‐3′ and 5′‐TGGTCCAGGGTTTCTTACTCC‐3′; human *AhR*: 5′‐TTGGTTGTGATGCCAAAGGAAG‐3′ and 5′‐CGGATATGGGACTCGGCAC‐3′; human *CYP1A1*: 5′‐AGACACAGTGATTGGCAGGTC‐3′ and 5′‐TGTCTCTTGTTGTGCTGTGGG‐3′; human *CYP1B1*: 5′‐CTCCTCCTCTTCACCAGGTATC‐3′ and 5′‐GGCTGGTCACCCATACAAGG‐3′; human *IL‐8*: 5′‐CTCCAAACCTTTCCACCCCA‐3′ and 5′‐TTCTCAGCCCTCTTCAAAAACTTC‐3′; human *TNF‐α*: 5′‐CCCATCTATCTGGGAGGGGT‐3′ and 5′‐GCGTTTGGGAAGGTTGGATG‐3′; human *IL‐1β*: 5′‐AGGCTGCTCTGGGATTC‐3′ and 5′‐GCCACAACAACTGACGC‐3′; human *GAPDH*: 5′‐GGAGCGAGATCCCTCCAAAAT‐3′ and 5′‐GGCTGTTGTCATACTTCTCATGG‐3′.

### 2.15 Western Blotting

The collected colon tissue of mice was lysed with RIPA lysate to extract total protein. For cellular protein samples, low‐temperature lysis was performed with RIPA lysis buffer according to the manufacturer's instructions, and total protein samples were obtained by centrifugation at 4°C. Protein samples were separated by 10% SDS‐PAGE, and the target proteins were transferred onto a 0.45 μm PVDF membrane, which was then blocked with 5% skimmed milk powder at room temperature, incubated with primary antibody (AhR Monoclonal antibody, 67,785–1‐Ig; CYP1A1 Polyclonal antibody, 13,241–1‐AP; CYP1B1 Monoclonal antibody, 67,033–1‐Ig; Proteintech, China) at 4°C overnight. Following a one‐hour incubation at room temperature with a horseradish peroxidase‐conjugated secondary antibody, the membrane was subsequently analyzed using an enhanced chemiluminescence reagent. For normalization, GAPDH or β‐actin was used as the loading control, depending on the molecular weight of the target protein. The relative protein expression of each target was normalized to the corresponding internal control signal.

### 2.16 Data Analysis

All values are presented as mean values with their corresponding standard error of the mean (SEM). Differences between two independent groups were assessed using unpaired Student's *t*‐test, while comparisons involving three or more groups were performed using one‐way ANOVA followed by Tukey's HSD post hoc test for pairwise multiple comparisons. SPSS version 26.0 was employed for all statistical analyses, with a significance threshold set at *p* values less than 0.05. For differential abundance analysis of 16S rRNA sequencing data, taxa with significantly different relative abundances between groups were identified using [LEfSe/Wilcoxon rank‐sum test]. *p*‐values were adjusted for multiple comparisons using the Benjamini‐Hochberg false discovery rate (FDR) method. Taxa with FDR‐adjusted *q* < 0.05 were considered statistically significant.

## 3 Results

### 3.1 Identification of the Components of BTWT

The chemical constituents of BTWT were analyzed using UHPLC‐HRMS. Figure 1A1–B2 shows the total ion chromatogram and base peak chromatogram in the positive and negative ion modes. The chemical constituents of BTWT include alkaloids, phenylpropanoids, sugars and glycosides, terpenoids, organic acids and their derivatives and others, among which alkaloids, phenylpropanoids, and terpenoids are the main chemical constituents Figure 1C. Figure 1D1–G2 shows the EIC and MS spectra of the more abundant chemical components in BTWT, including Pulsatilla saponin D, Pulchinenoside B, 23‐Hydroxybetulinic acid, Oleanolic Acid, Berberine, Oxyberberine, 8‐Oxocoptisine, Obacunone, Esculin, Esculetin, and Fraxetin. Table 4 is the detailed information of LC–MS characterization of the main components of BTWT, including retention time, molecular formula, theoretical and actual m/z values, fragment ions and other information.

**FIGURE 1:**
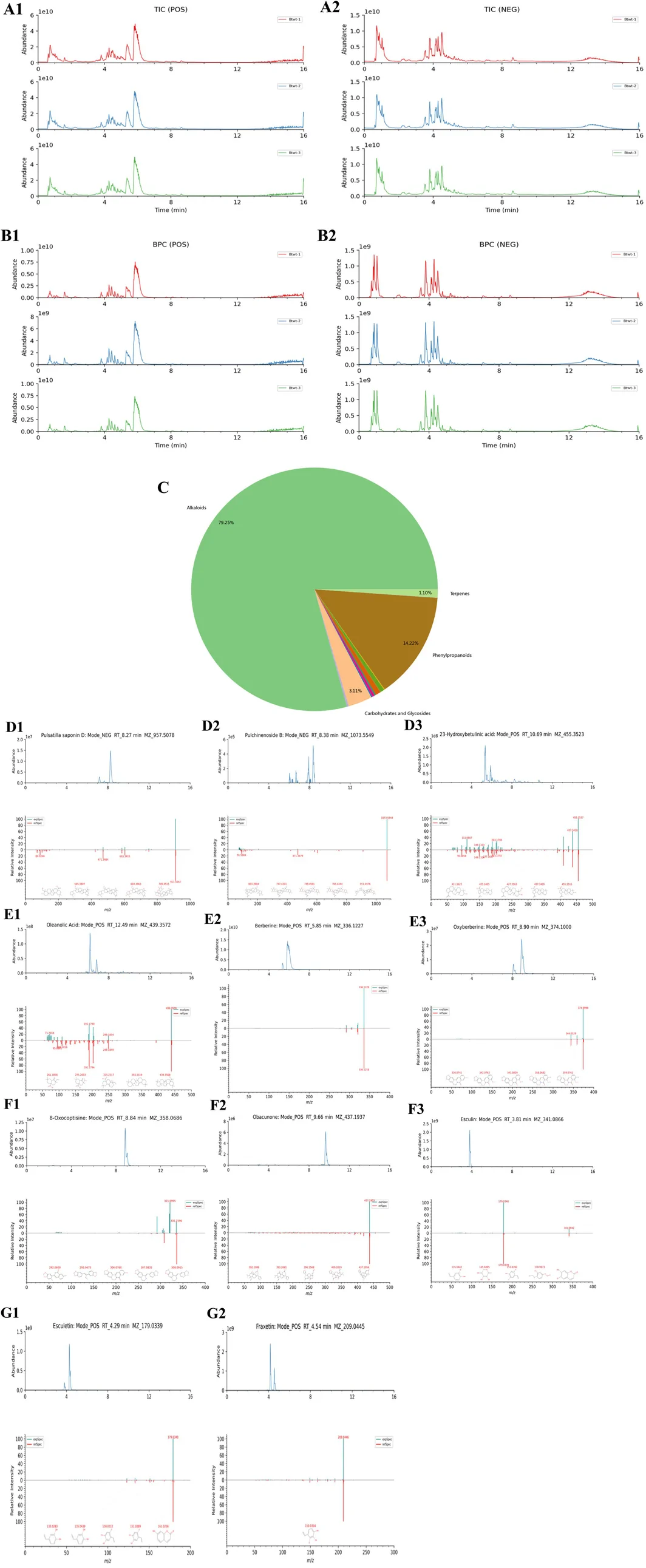
Identification of BTWT Components. (A1) TIC plot in positive ion mode; (A2) TIC plot in negative ion mode; (B1) BPC plot in positive ion mode; (B2) BPC plot in negative ion mode; (C) Distribution of categorical BTWT components; (D1) EIC and MS spectra of Pulsatilla saponin D; (D2) EIC and MS spectra of Pulchinenoside B; (D3) EIC and MS spectra of 23‐Hydroxybetulinic acid; (E1) EIC and MS spectra of Oleanolic Acid; (E2) EIC and MS spectra of Berberine; (E3) EIC and MS spectra of Oxyberberine; (F1) EIC and MS spectra of 8‐Oxocoptisine; (F2) EIC and MS spectra of Obacunone; (F3) EIC and MS spectra of Esculin; (G1) EIC and MS spectra of Esculetin; (G2) EIC and MS spectra of Fraxetin.

**TABLE 4:** LC–MS characterization of main chemical components in BTWT.

Source of medicinal materials	Compound name	Retention time	Molecular formula	Theoretical m/z	m/z	Fragment ions	Error (ppm)	Chemical composition category
Bai Tou Weng	Pulsatilla saponin D	8.27	C_47_H_76_O_17_	957.5064	957.5078	471.3485, 585.3803, 603.3906, 911.501	1.47	Terpenoids
Bai Tou Weng	Pulchinenoside B	8.38	C_53_H_86_O_22_	1073.5538	1073.5549	74.3574, 74.4782, 74.5085, 77.585, 77.6943, 1073.5544	0.99	Terpenoids
Bai Tou Weng	23‐Hydroxybetulinic acid	10.69	C_30_H_48_O_4_	455.352	455.3523	201.1639, 203.1798, 207.1747, 215.1798, 219.1747, 409.3473, 425.3430, 437.3418, 455.2144, 455.3537	0.67	Terpenoids
Bai Tou Weng	Oleanolic Acid	12.49	C_30_H_48_O_3_	439.3571	439.3572	77.7522, 85.1599, 95.0861, 109.1013, 189.1641, 191.1790, 203.1789, 205.1944, 249.1854, 439.3576	0.34	Terpenoids
Huang Lian	Berberine	5.85	C_20_H_18_NO_4_	336.1236	336.1227	292.0968, 320.0921, 321.0996, 336.1229	−2.51	Alkaloids
Huang Lian	Oxyberberine	8.9	C_20_H_17_NO_5_	374.0999	374.1	344.0529, 359.0762, 374.0998	0.24	Alkaloids
Huang Lian	8‐Oxocoptisine	8.84	C_19_H_13_NO_5_	358.0686	358.0686	292.0967, 304.0953, 306.0766, 308.0925, 320.092, 321.0995, 335.2196	0.05	Alkaloids
Huang Bai	Berberine	5.85	C_20_H_18_NO_4_	336.1236	336.1227	292.0968, 320.0921, 321.0996, 336.1229	−2.51	Alkaloids
Huang Bai	Obacunone	9.66	C_26_H_30_O_7_	437.1958	437.1937	437.1933	−4.82	Terpenoids
Qin Pi	Esculin	3.81	C_15_H_16_O_9_	341.0867	341.0866	179.034, 341.0842	−0.32	Phenylpropanoids
Qin Pi	Esculetin	4.29	C_9_H_6_O_4_	179.0339	179.0339	179.034	0.12	Phenylpropanoids
Qin Pi	Fraxetin	4.54	C_10_H_8_O_5_	209.0444	209.0445	209.0446	0.22	Phenylpropanoids

### 3.2 Successful Establishment of Microbial Depletion Mouse Model

In this study, a microbial depletion mouse model was established using an antibiotic gavage. The findings of this study indicated that the administration of antibiotics had no significant effect on the body weight changes of the mice (Figure S1A), whereas the spleen weight and gross changes in the cecum of the mice were consistent with the characteristics of the microbial depletion mouse model (Figure S1B,C). Next, we analyzed the 16SrRNA sequencing of mouse cecum contents and found that the α‐diversity (including Chao, Ace, Sob, and Shannon) of the gut microbiota was significantly reduced by antibiotic treatment (Figure S1D1–4). However, the β‐diversity (including PCA, PCoA, and NMDS) (Figure S1E1–3) indicated that the mouse gut microbiota changed significantly after antibiotic treatment compared to that in the control group. At the phylum level (Figure S1F1), the control group had a higher abundance of Bacteroidetes, Firmicutes, and Actinobacteria, whereas the antibiotic‐treated group had only one phylum, Proteobacteria. At the genus level (Figure S1G1), the control group had a high abundance of *norank_f _muribaculaceae*, *Lactobacillus*, *Lachnospiraceae _nk4a136_group*, *Coriobacteriaceae_ucg‐002*, *unclassified_f*_ *ruminococcaceae*, *unclassified f lachnospiraceae, and dubosiella*, whereas the antibiotic‐treated group had only the more homogeneous *Enterobacter* and *escherichia‐shigella*. Figure S1F2,G2 demonstrate the significance of intergroup differences at the phylum and genus levels, respectively, and the differences were highly significant (*p* < 0.001) for both the phyla and genera illustrated in the Figures. These findings were consistent with those of a pseudo‐bacteria‐free mouse model (Reikvam et al. 2011; Zarrinpar et al. 2018).

**FIGURE 2:**
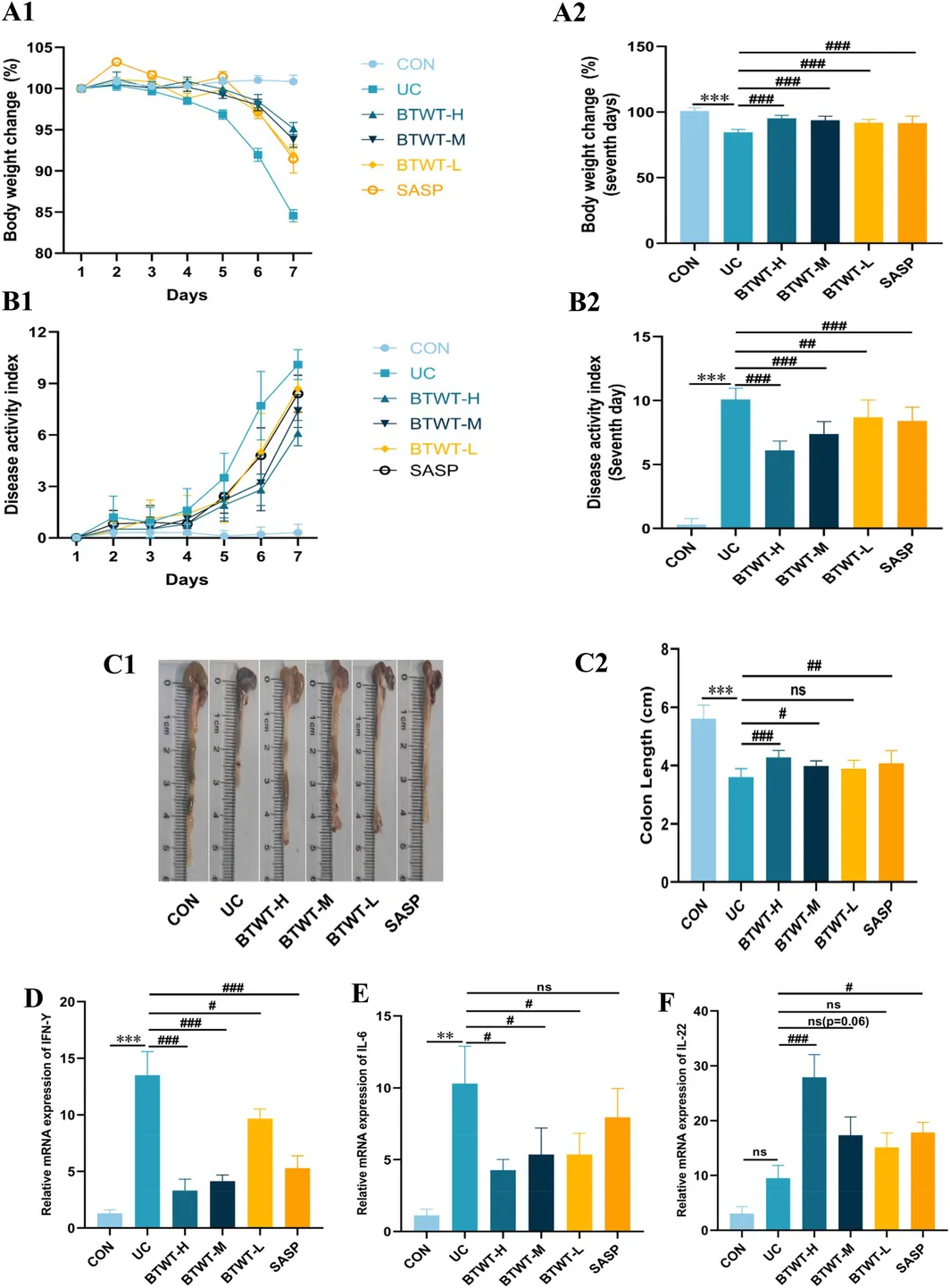
BTWT alleviates the symptoms of colitis mice. (A1) Weight change rate during the experiment; (A2) weight change rate on the seventh day; (B1) DAI score during the experiment; (B2) DAI score on the seventh day; (C1–2) colon length; (D) *Ifn‐γ* mRNA; (E) *Il‐6* mRNA; (F) *Il‐22* mRNA. Data are expressed as the mean ± SEM, *p < 0.05, **p < 0.01, ***p < 0.001 indicates the UC group compared with the CON group; ^#^ *p* < 0.05, ^##^ *p* < 0.01，^###^ *p* < 0.001 indicates the treatment group compared with the UC group; ^ns^ *p* indicates no difference.

### 3.3 BTWT Alleviates the Symptoms of Colitis Mice

Unlike the CON group, the UC model mice exhibited markedly lower body weight but higher DAI scores. High, medium, and low doses of BTWT and SASP could effectively inhibit weight loss (Figure 2A1–2) and decrease DAI score (Figure 2B1–2). Unlike the CON group, the mice with DSS‐induced colitis showed a marked shortening in colon length, whereas both BTWT at varying dosages and SASP managed to curb this shortening to different extents (Figure 2C1–2). Colon tissue from the UC group exhibited substantially elevated *Ifn‐γ* and *Il‐6* mRNA levels compared to the CON group. Following BTWT and SASP treatments at varied dosages, *Ifn‐γ* and *Il‐6* mRNA expression diminished differentially (Figure 2D,E). Moreover, BTWT and SASP administration differentially elevated *Il‐22* mRNA expression across tested dosage levels (Figure 2F). Therefore, BTWT can effectively alleviate the inflammatory injury of colitis mice and has a fine protective effect on colitis mice.

### 3.4 BTWT Improves Gut Microbiota Composition and Variety in Murine Colitis Models

Figure 3A1–3 illustrates a marked shift in the α‐diversity of the gut microbiota among the mice in the model group, as opposed to the CON group. Following the BTWT intervention, however, all indices exhibited varying degrees of recovery. Meanwhile, β‐diversity analysis revealed substantial shifts in the bacterial composition across study groups, with the model group showing greater divergence from the CON group compared to the BTWT intervention group, which clustered more closely with the CON group (Figure 3B1–3,C). The Venn diagram indicated 460 OTUs across the three groups (Figure 3D). The UC group saw a surge in Bacteroidetes, Proteobacteria, and Desulfobacterota populations at the phylum level when stacked up against the CON group, whereas Firmicutes took a nosedive; the BTWT intervention group managed to turn the tables on this pattern (Figure 3E,F1–2). When examining the genus level, the model group showed a notable uptick in *Bacteroides*, *norank_f_desulfovibrionaceae*, and *Alistipes* compared to the CON group. On the flip side, *Bifidobacterium* and *Dubosiella* took a nosedive in relative abundance. Interestingly enough, the BTWT group saw a different pattern altogether, with *Bifidobacterium*, *Turicibacter*, *Dubosiella*, and *Alistipes* all getting a boost in their numbers (Figure 3G,H1–7).

**FIGURE 3:**
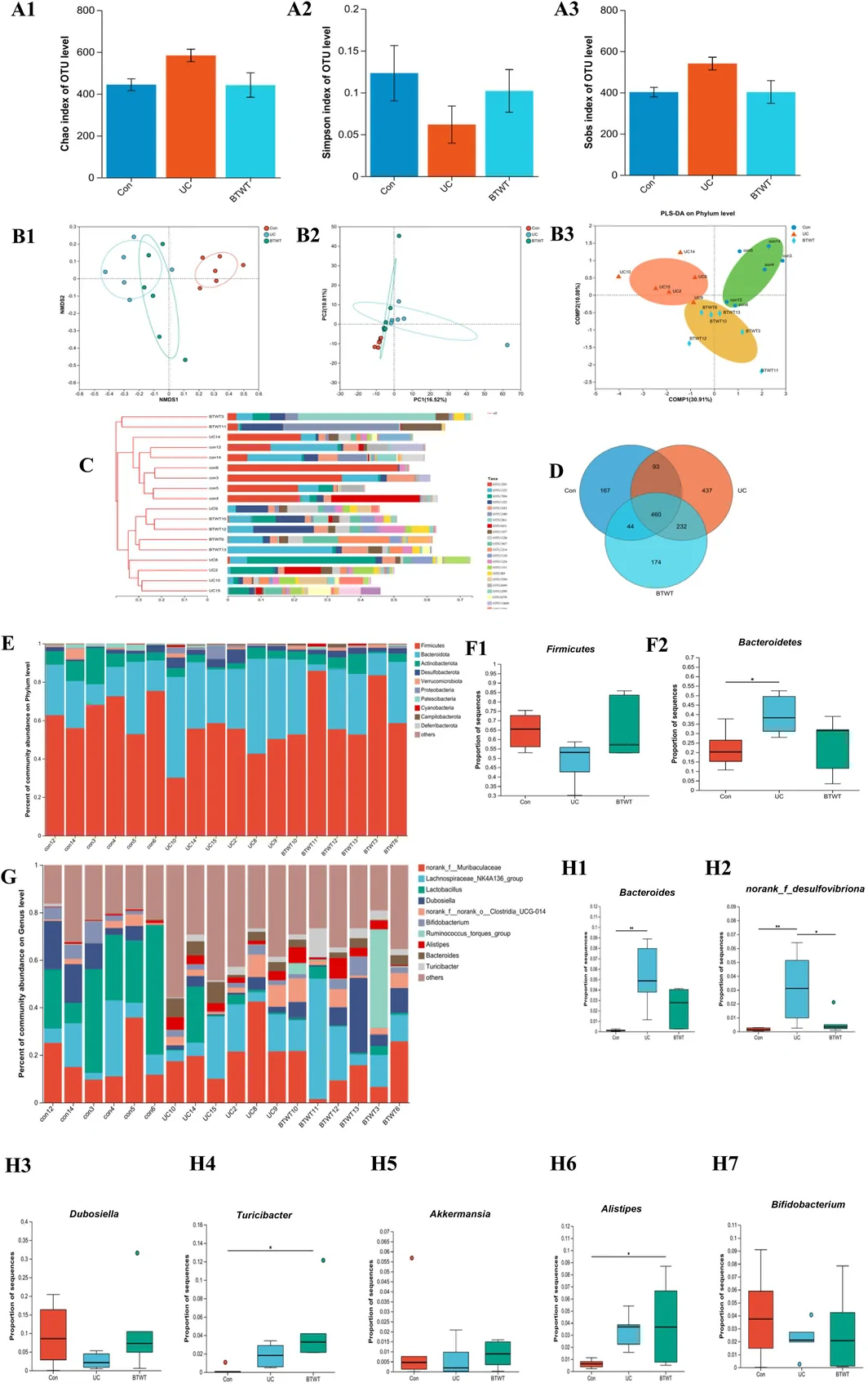
BTWT improves gut microbiota composition and variety in murine colitis models. (A1) Chao index; (A2) Simpson index; (A3) Sobs index; (B1) NMDS; (B2) PCoA; (B3) PLS–DA; (C) hierarchical cluster analysis; (D) OTU‐based Venn diagram. (E) Changes in the gut microbiota of mice at the phylum level; (F1–2) Intergroup significance test of flora with significant changes in abundance at the phylum level. (G) Changes in mouse gut microbiota at the genus level. (H1–7) Test of significance between groups of bacteria with significant changes in abundance at the genus level.

### 3.5 BTWT Relieves Colitis in Mice in a Gut Microbiota‐Dependent Manner

We used antibiotics to remove intestinal microbiome (Figure 4A). The results showed that compared with the CON group, mice in the UC model group had decreased body weight (Figure 4B1–2), and DAI scores were significantly higher (Figure 4C1–2), and colon length was significantly shortened (Figure 4D). The colon's histopathological assessment revealed markedly elevated scores (Figure 4K1), characterized by submucosal edema and thickening, along with a severely compromised mucosal architecture. This manifested as extensive inflammatory cell infiltration, crypts dissolved and disappeared, and a significant depletion of goblet cells throughout the tissue (Figure 4I,J); colonic tissue exhibited significantly increased *Ifn‐γ* and *Il‐6* mRNA expression, accompanied by decreased *Il‐22* mRNA levels (Figure 4L1–3). After the BTWT intervention, all indices showed different degrees of improvement, which manifested as inhibition of weight loss, reduction of DAI scores, inhibition of colon shortening, reduction of histopathological scores, reduction of *Il‐6* and *Ifn‐γ* mRNA expression, and promotion of mRNA expression of *Il‐22* mRNA. However, after antibiotic treatment, the beneficial effect of BTWT on colitis was greatly weakened. Immediately, we performed FMT (Figure 4E). FMT from BTWT‐treated donors partially restored the protective effects of BTWT, as evidenced by FMT‐BTWT ameliorated colitis manifestations, encompassing body mass fluctuations and DAI (Figure 4F,G), reduced colitis infiltration and mucosal damage, restored colon tissue structure and goblet cell numbers (Figure 4I,J), while FMT without BTWT intervention (FMT‐UC) showed the opposite results. In addition, FMT‐BTWT suppressed *Ifn‐γ* and *Il‐6* mRNA expression in colon tissue while significantly upregulated *Il‐22* mRNA levels (Figure 4M1–3). Although the degree of restoration was incomplete compared to direct BTWT treatment, FMT‐BTWT still achieved a marked improvement over the FMT‐UC.

**FIGURE 4:**
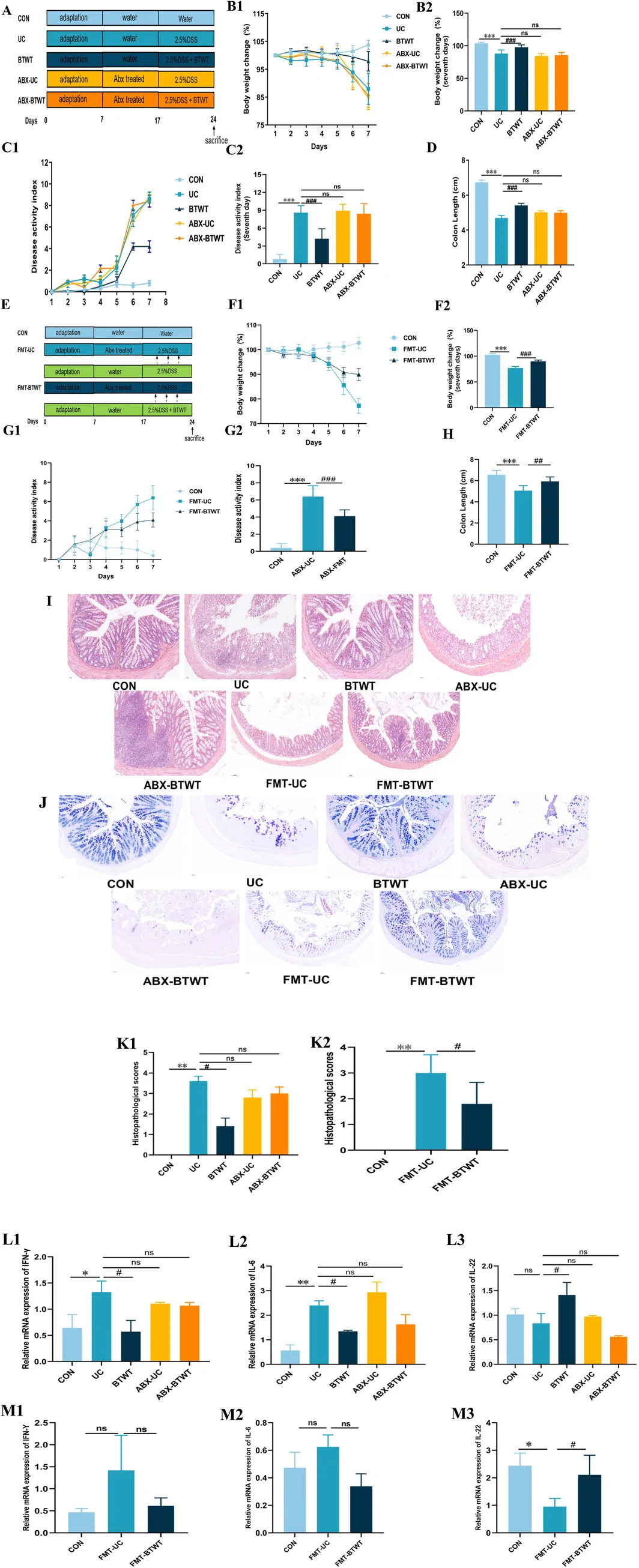
BTWT alleviates colitis in mice in a gut microbiota‐dependent manner. (A) ABX depletion of intestinal microbial schematic diagram; (B1) Weight change rate during ABX experiment; (B2) Body weight change rate on the seventh day of ABX; (C1) DAI score during ABX experiment; (C2) ABX seventh day DAI score; (D) ABX colon length; (E) FMT schematic diagram; (F1) Weight change rate during FMT experiment; (F2) Body weight change rate on the seventh day of FMT; (G1) DAI score during FMT experiment; (G2) DAI score on the seventh day of FMT; (H) FMT colon length; (I) HE staining of colon tissue; (J) AB‐PAS staining of colon tissue. (K1) ABX histopathological injury score; (K2) FMT histopathological injury score; (L1–3) ABX *Ifn‐γ*, *Il‐6*, *Il‐22* mRNA; (M1–3) FMT *Ifn‐γ*, *Il‐6*, *Il‐22* mRNA. Data are expressed as the mean ± SEM, *p < 0.05, **p < 0.01, ***p < 0.001 indicates the UC group compared with the CON group; ^#^ *p* < 0.05, ^##^ *p* < 0.01，^###^ *p* < 0.001 indicates the treatment group compared with the UC group; ^ns^ *p* indicates no difference.

### 3.6 BTWT Regulates Tryptophan Metabolism in Colitis Mice in a Gut Microbe‐Dependent Manner

Table 5 details of tryptophan metabolites LC–MS/MS. As shown in the PLS‐DA and PCA plots, Figure 5A1–2 show good separation between the groups. The BTWT intervention cohort was more akin to the control group, whereas the other three sets were notably more distant. This suggests that the disparity between the BTWT group and the CON group was notably reduced. The findings from the expression pattern cluster analysis bore this out. Figure 5B1–2 showed that the trends of changes in substance expression in the CON and intervention groups of BTWT were similar, while the remaining three groups were similar. Figure 5C illustrates the distribution profile of tryptophan metabolites, aligning with previous findings where the BTWT intervention group's substance levels nearly mirrored those of the CON group. In contrast, while the other three groups showed similar patterns, they fell short of the BTWT group's abundance. DSS treatment reduced the contents of 5‐Hydroxyindole‐3‐acetic acid, Indole‐3‐acetic acid, 3‐Hydroxy‐DL‐kynurenine, 3‐Indoleacetonitrile, Nicotinic Acid, Kynurenic Acid, and Xanthurenic acid in feces. The contents of some metabolites increased after BTWT intervention, such as Indole, Indole‐3‐lactic Acid, 5‐Hydroxyindole‐3‐acetic acid, 5‐hydroxytryptophan, 3‐Hydroxy‐DL‐kynurenine, Nicotinic Acid, N‐Acetyl‐serotonin, L‐Tryptophan, Kynurenic Acid, and Serotonin. Xanthurenic acid, in which the metabolites with significant differences were Indole, Indole‐3‐lactic Acid, 3‐Hydroxy‐DL‐kynurenine, and Serotonin, and the content of other metabolites increased but there was no significant difference (Figure 5D1–H2).

**TABLE 5:** LC–MS/MS details of tryptophan metabolism.

No.	Metabolite name	Precursor ion	Product ion	Retention time	Declustering potential	Collision energy
1	Indole	118.2	91	13.39	140	32
2	Indole‐3‐lactic Acid	206.2	118.2	9.14	35	30
3	5‐Hydroxyindole‐3‐acetic acid	192.1	146.2	7.38	50	16
4	Indole‐3‐acetic acid	176.2	130.2	10.44	40	35
5	5‐hydroxytryptophan	221.2	204	3.94	20	15
6	3‐Hydroxy‐DL‐kynurenine	225.1	208.1	3.75	18	13
7	Nicotinamide (Vitamin B3)	123.1	80	2.74	40	28
8	3‐Indoleacetonitrile	157.2	130.2	12.7	50	18
9	Nicotinic Acid	124.1	79.9	2.33	65	25
10	3‐hydroxyanthranilic acid	154.2	136.1	5.14	30	19
11	N‐Acetyl‐serotonin	219.2	160.1	7.78	30	20
12	L‐Tryptophan	205.1	146.1	5.48	80	25
13	Kynurenine	209.1	174.2	4.2	20	22
14	Kynurenic Acid	190.1	144.2	6.45	60	26
15	Tryptamine	161.1	144.2	6.52	10	16
16	Serotonin	177.2	160.2	3.94	10	16
17	Tryptophol	162.1	117.3	10.53	30	31
18	Xanthurenic acid	206.1	160.1	5.52	60	25

**FIGURE 5:**
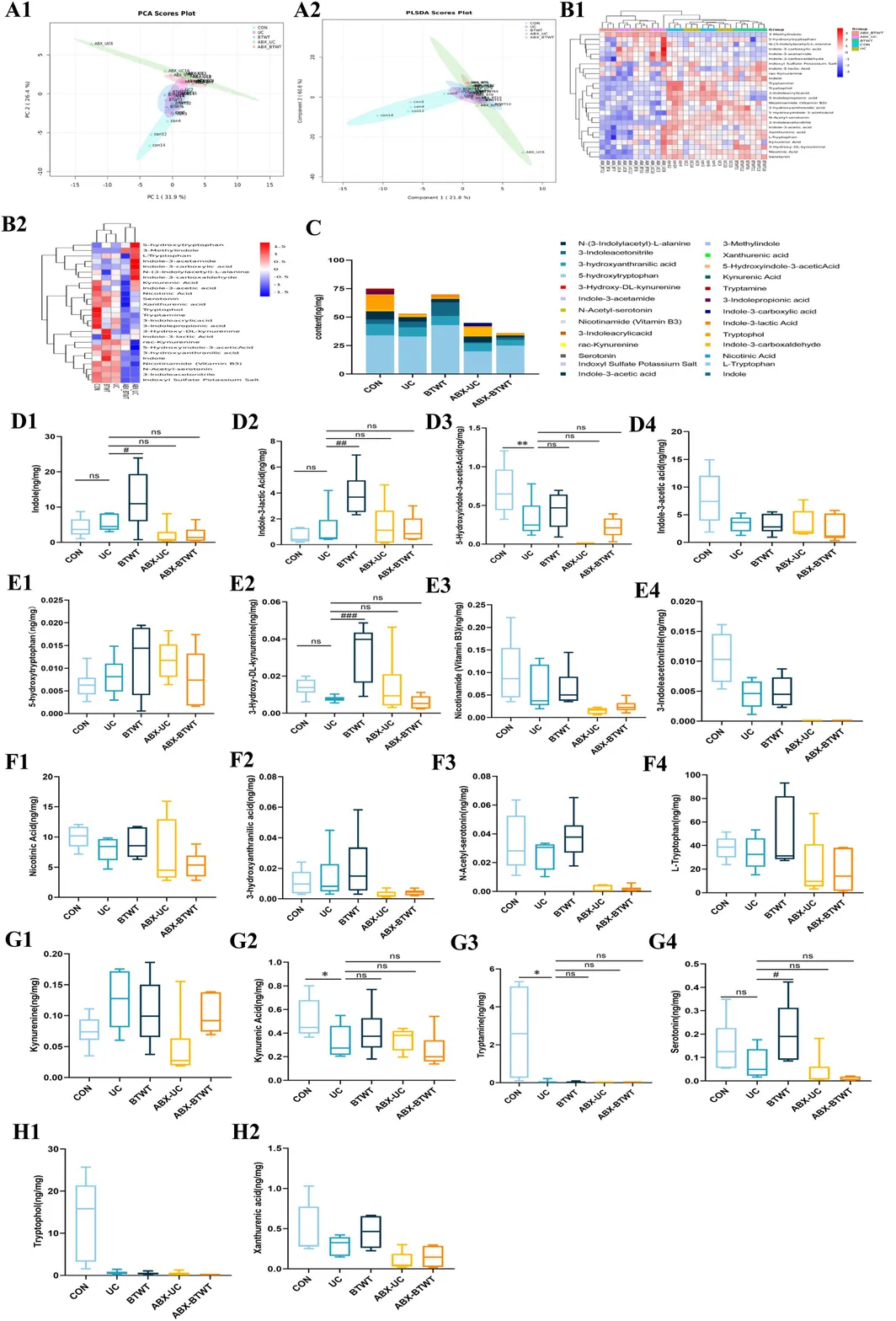
Metabolic analysis of mouse fecal tryptophan. (A1) PCA plot; (A2) PLS‐DA plot; (B1–2) clustered heatmap of expression patterns; (C) histogram of the relative abundance of each metabolite; (D1) Indole; (D2) Indole‐3‐lactic Acid; (D3) 5‐Hydroxyindole‐3‐acetic acid; (D4) Indole‐3‐acetic acid; (E1) 5‐hydroxytryptophan; (E2) 3‐Hydroxy‐DL‐kynurenine; (E3) Nicotinamide (Vitamin B3); (E4) 3‐Indoleacetonitrile; (F1) Nicotinic Acid; (F2) 3‐hydroxyanthranilic acid; (F3) N‐Acetyl‐serotonin; (F4) L‐Tryptophan; (G1) Kynurenine; (G2) Kynurenic Acid; (G3) Tryptamine; (G4) Serotonin; (H1) Tryptophol; (H2) Xanthurenic acid. Data are expressed as the mean ± SEM, *p < 0.05, **p < 0.01 indicates the UC group compared with the CON group; ^#^ *p* < 0.05, ^##^ *p* < 0.01; ^###^ *p* < 0.001 indicates the treatment group compared with the UC group；^ns^ *p* indicates no difference.

### 3.7 Correlation Analysis Between Significantly Different Microbiota and Metabolites

To get to the bottom of the complex relationships between microbial communities and metabolites in our experimental samples, we turned to Spearman's rank correlation analysis paired with hierarchical clustering in R. This multi‐pronged approach allowed us to connect the dots between the flora and metabolite interactions from various angles, as illustrated in Figure 6. Indole‐3‐lactic acid was significantly positively correlated with *Turicibacter* and *Romboutsia*, and significantly negatively correlated with *Prevotellaceae_Ga6A1_group*; indole‐3‐acetic acid was significantly positively correlated with *Dubosiella* and *Monoglobus* and significantly positively correlated with *Alistipes*, *Erysipelatoclostridium*, and *Faecalitalea*; indole‐3‐acrylic acid was significantly positively correlated with *Lactobacillus*, *Prevotellaceae_Ga6A1_group*, and *Monoglobus*, and significantly negatively correlated with *Turicibacter* and *Romboutsia*; indole was significantly positively correlated with *Romboutsia*.

**FIGURE 6:**
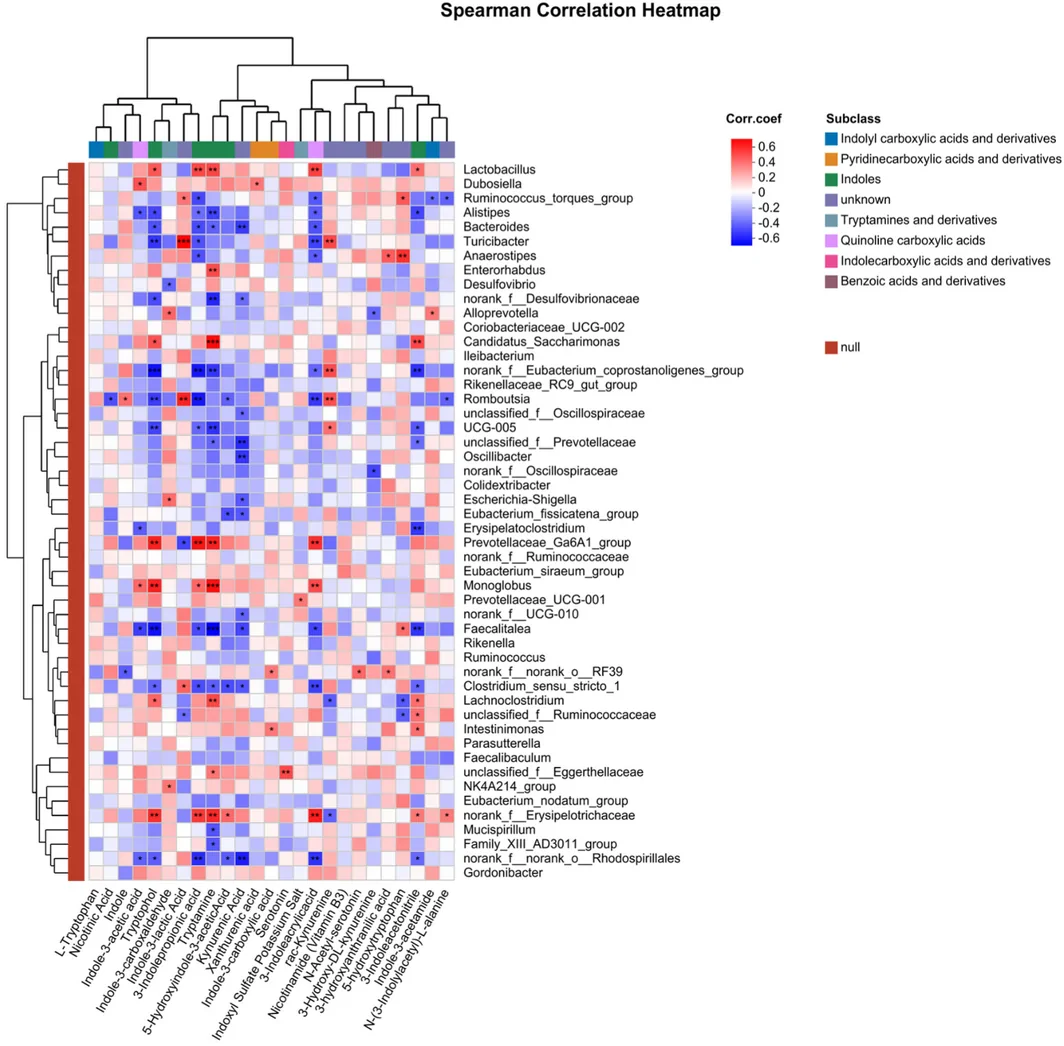
Correlation analysis of significantly different flora with significantly different metabolites. The microbial classification is shown on the right side of the heat map, and the metabolites are shown below. Clustering dendrograms based on correlation coefficients are constructed on the left and top, respectively. The color bar represents the magnitude of the correlation coefficient: The closer the absolute value is to 1, the higher the metabolite correlation; red indicates a positive correlation, blue indicates a negative correlation, * *p* < 0.05, **p < 0.01, ***p < 0.001.

### 3.8 BTWT Depends on Gut Microbes to Restore AhR Expression in Colitis Mice

As shown in Figure 7A1–B3, compared with the CON group, the protein content and mRNA level of *Ahr*, *Cyp1a1*, and *Cyp1b1* in the colitis model group decreased, while BTWT intervention significantly increased the relative expression of AhR, CYP1A1, and CYP1B1 protein and mRNA. After antibiotic treatment, the effect of BTWT was severely weakened (Figure 7A1–B3). FMT from BTWT‐treated donors partially restored the expression of AhR in colon tissue, as evidenced by FMT‐BTWT markedly upregulating *Ahr* and downstream genes for *Cyp1a1* and *Cyp1b1* at both protein and mRNA levels (Figure 7C1–D3).

**FIGURE 7:**
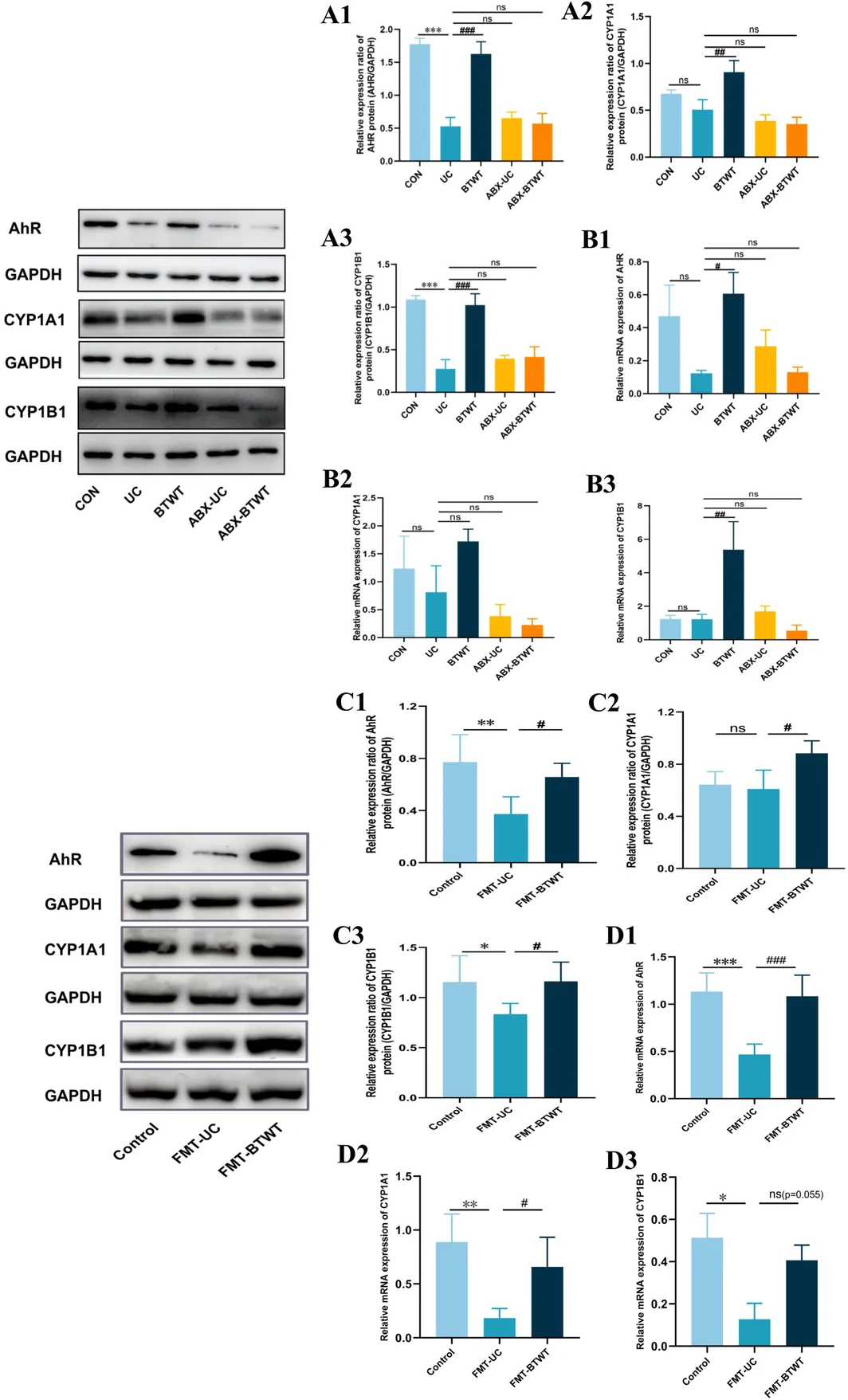
BTWT depends on gut microbes to restore the expression of AhR in mice with colitis. (A1) and (C1) Relative expression of AhR protein; (A2) and (C2) Relative expression of CYP1A1 protein; (A3) and (C3) Relative expression of CYP1B1 protein; (B1) and (D1) Relative mRNA expression of *Ahr*; (B2) and (D2) Relative mRNA expression of *Cyp1a1*; (B3) and (D3) Relative mRNA expression of *Cyp1b1*. Data are expressed as the mean ± SEM, *p < 0.05, **p < 0.01; ***p < 0.001 indicates the UC group compared with the CON group; ^#^ *p* < 0.05, ^##^ *p* < 0.01; ^###^ *p* < 0.001 indicates the treatment group compared with the UC group; ^ns^ *p* indicates no difference.

### 3.9 Tryptophan Metabolite ILA Attenuates HT‐29 Cell Inflammation Through AhR Pathway Involvement

In order to explore the mechanism of BTWT regulating AhR, we studied the specific effect mechanism of BTWT and ILA on the activation of AhR in intestinal epithelial cells. As shown in Figure 8, BTWT and ILA acted on HT‐29 cells for 12 h (Figure 8A1–2) and 24 h (Figure 8C1–2), respectively, and BTWT did not affect the viability of cells at 640 μg/mL and below. ILA did not affect cell viability at 25 μM and below. Immediately following this, we analyzed the protein expression of AhR, and the results showed that ILA significantly elevated the relative expression of AhR protein after 12 h and 24 h of action, respectively. In contrast, BTWT did not affect the AhR protein expression (Figure 8B,D). We further analyzed the effect of BTWT on the protein expression of AhR target proteins CYP1A1 and CYP1B1 and found that BTWT had no significant effect on the protein expression of CYP1A1 and CYP1B1 (Figure 8E1–3). Therefore, we believe that the ability of BTWT to regulate and activate AhR is attributable to its indirect effect on tryptophan metabolism rather than its direct impact. Next, an in vitro HT‐29 cell inflammation model was used to evaluate whether ILA has anti‐inflammatory activity. In this study, we used different concentrations of lipoteichoic acid (LTA) to stimulate HT‐29 cells for 6 h, 12 h, 24 h. As shown in Figure 9A1–D3, we determined that the optimal modeling condition was 5 μg/mL LTA stimulation of HT‐29 cells for 6 h. We assessed ILA's anti‐inflammatory impact and cellular pathway in this experimental model. Figure 10A1–3 illustrates that following exposure to various concentrations of ILA (10, 5, and 2.5 μM), the mRNA expression levels of *IL‐8*, *TNF‐α*, and *IL‐1β* experienced a marked reduction. Immediately after this, we examined the relative expression of *AhR*, *CYP1A1*, and *CYP1B1* mRNA and protein. The results revealed that the mRNA levels of *AhR*, *CYP1A1*, and *CYP1B1* significantly decreased after modeling (*p* < 0.05), whereas 5 μM ILA significantly restored the protein levels of AhR, CYP1A1, and CYP1B1 (Figure 10B1–3). Likewise, 5 μM ILA markedly increased mRNA levels of *AhR*, *CYP1A1*, and *CYP1B1* (Figure 10C1–3).

**FIGURE 8:**
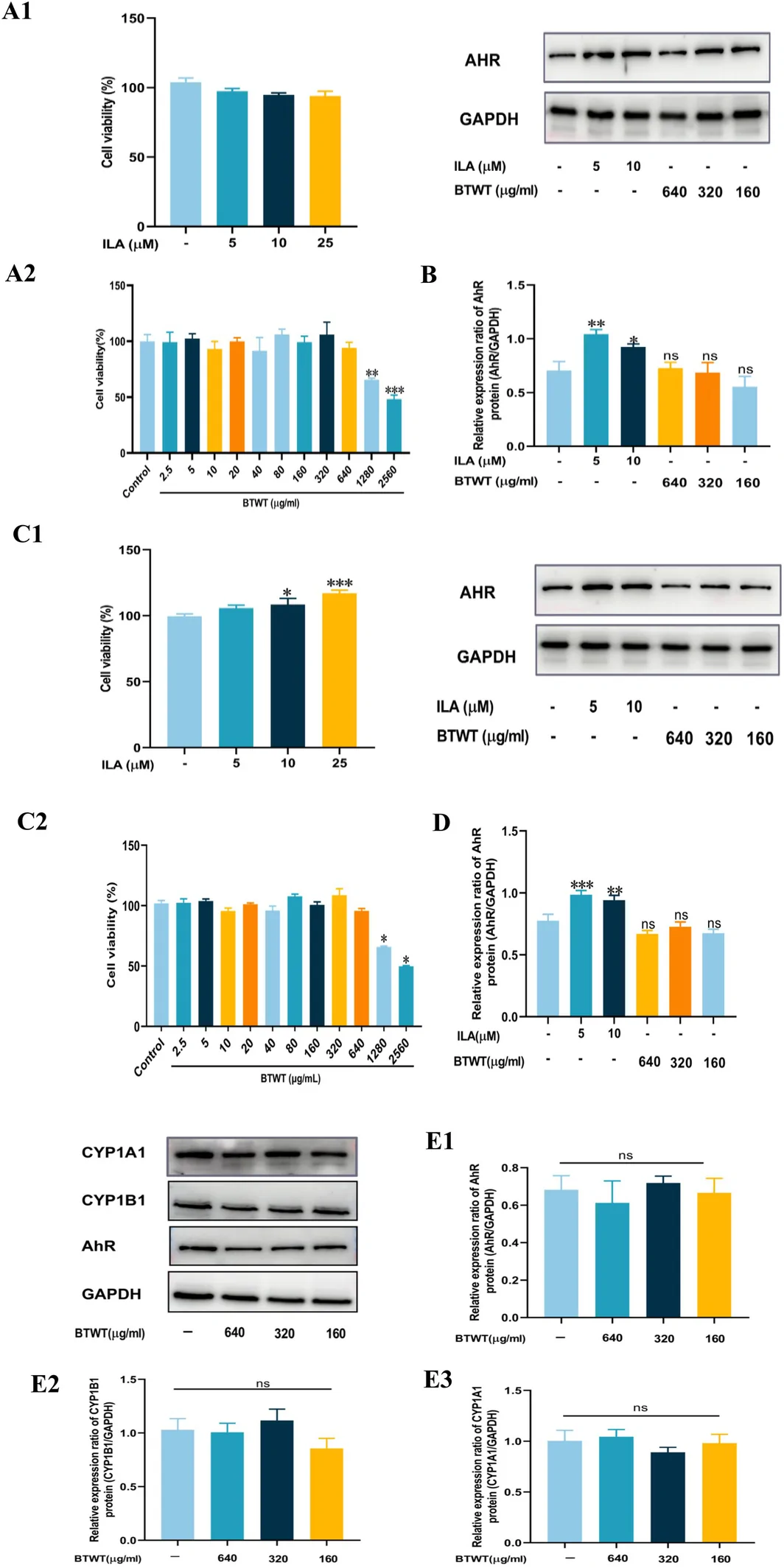
BTWT activates AhR by regulating ILA. (A1) and (A2) are the changes in cell viability of HT‐29 cells after 12 h of ILA and BTWT; (B) the changes in AhR protein expression after 12 h of ILA and BTWT action in HT‐29 cells; (C1) and (C2) are the changes in cell viability of HT‐29 cells after 24 h of ILA and BTWT; (D) the changes in AhR protein expression after 24 h of ILA and BTWT action in HT‐29 cells; (E1–3) changes in AhR, CYP1A1 and CYP1B1 protein expression after 24 h of BTWT action in HT‐29 cells. Data are expressed as mean ± SEM, *p < 0.05, **p < 0.01, ***p < 0.001 indicates the treated group compared with the control group; ^ns^ *p* indicates no difference.

**FIGURE 9:**
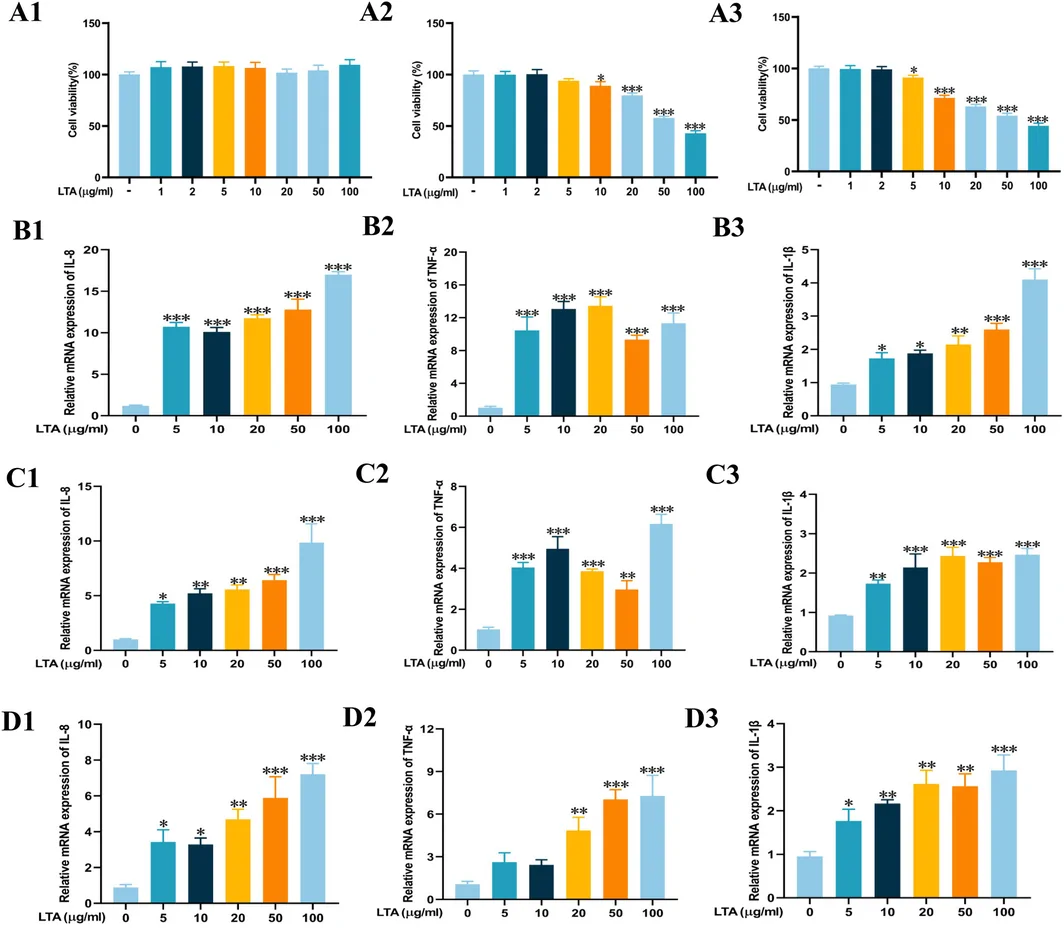
LTA‐induced HT‐29 cell inflammation model. (A1–3) Cell viability assay of HT‐29 cells stimulated with LTA for 6 h, 12 h, and 24 h; (B1–3) The mRNA of *IL‐8*, *TNF‐α*, and *IL‐1β* in HT‐29 cells after 6 h of stimulation with LTA; (C1–3) The mRNA of *IL‐8*, *TNF‐α*, and *IL‐1β* in HT‐29 cells after 12 h of stimulation with LTA; (D1–3) The mRNA of *IL‐8*, *TNF‐α*, and *IL‐1β* in HT‐29 cells after 24 h of stimulation with LTA. Data are expressed as mean ± SEM, *p < 0.05, **p < 0.01, ***p < 0.001 indicates the treated group compared with the control group.

**FIGURE 10:**
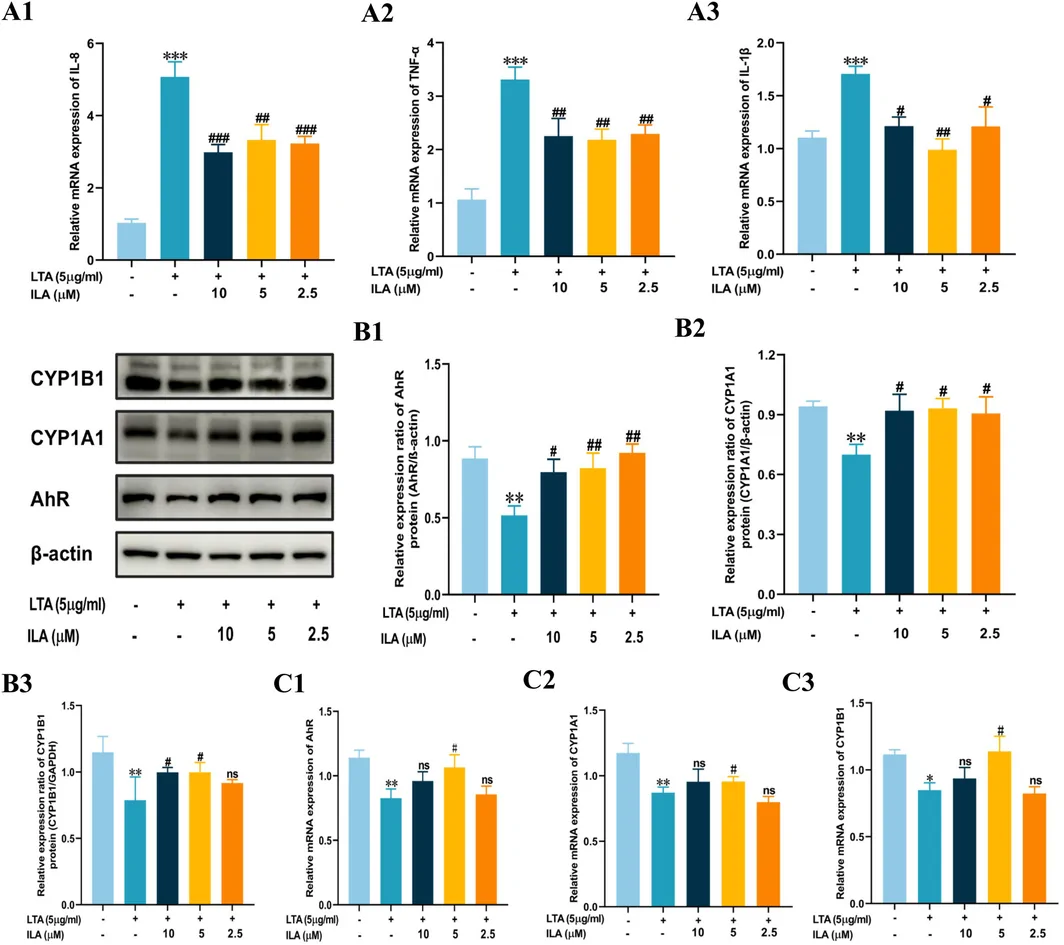
Tryptophan metabolite ILA attenuates HT‐29 cell inflammation through AhR pathway involvement. (A1–3) The mRNA expression changes of *IL‐8*, *TNF‐α*, and *IL‐1β*; (B1–3) The protein expression changes of AhR, CYP1A1, and CYP1B1; (C1–3) The mRNA expression changes of *AhR*, *CYP1A1*, and *CYP1B1*. Data are expressed as mean ± SEM, *p < 0.05, **p < 0.01; ***p < 0.001 indicates the model group compared with the control group; ^#^ *p* < 0.05, ^##^ *p* < 0.01; ^###^ *p* < 0.001 indicates the treated group compared with the model group; ^ns^ *p* indicates no difference.

## 4 Discussion

BTWT is a traditional Chinese medicine compound formula with a history of 1800 years and is widely used in clinical practice for the treatment of various gastrointestinal diseases, including diarrhea, bacillary dysentery, and ulcerative colitis (Xiao et al. 2024). The latest research shows that BTWT is a shield against harm to the lining of the colon, and it can also help improve ulcerative colitis triggered by DSS. It does this by tweaking the AMPK/mTOR signaling pathway, which in turn encourages autophagy; this process strengthens the junctions between cells (Pan et al. 2024). However, the relationship between BTWT and gut microbiota, gut microbiota tryptophan metabolites, and AhR signaling remains unclear. To investigate the above correlations, the present study induced a UC mouse model with DSS and administered BTWT intervention to investigate the anti‐inflammatory effects of BTWT and its effects on gut microbiota and tryptophan metabolism, and a pseudo‐bacterial mouse model was used to investigate the impact of gut microbiota on the anti‐inflammatory effects of BTWT, and whether BTWT activates the AhR directly was explored on HT‐29 cells in vitro. We found that BTWT regulated the gut microbiota, promoted tryptophan metabolism to activate AhR, and exerted a good anti‐UC effect. Interestingly, in a microbial depletion mouse model, the anti‐UC effect of BTWT was almost completely attenuated, and tryptophan metabolism was blocked, severely inhibiting AhR signaling activation, suggesting that BTWT is dependent on intestinal microbes to exert its anti‐UC effect. BTWT itself did not directly activate AhR; however, the tryptophan indole metabolites it modulated were associated with enhanced AhR expression.

The gut microbiota plays a crucial role in regulating host immunity, metabolism, signaling pathways, and other essential physiological functions, all of which are closely linked to the development of UC (Ni et al. 2017; Yu 2018). An increasing body of evidence suggests that a variety of gut microbiota, such as Bacteroidetes, Firmicutes, Proteobacteria, and Actinobacteria, are implicated in the onset and progression of UC. Meanwhile, an imbalance in the ratio of anamorphic and Firmicutes, which are the dominant phyla, often contributes to the occurrence of UC, with an increase in the relative abundance of Bacteroidetes and a decrease in the relative abundance of Firmicutes, which are characteristic features of UC development (Gao et al. 2023). The host‐gut flora connection typically exhibits a symbiotic nature, where the host nourishes the gut's microbial community. The gut microbiota has huge metabolic potential, resulting in the production of many metabolic small molecules, such as short‐chain fatty acids, bile acids, and tryptophan metabolites, which are essential for maintaining host health (Lloyd‐Price et al. 2019). Dysbiosis of the gut microbiota causes metabolic pathway disorders leading to a disproportionate ratio of relevant metabolites, which affects host intestinal immunity, damages the intestinal epithelium, and promotes the occurrence and development of UC. In the current research, the sequencing data revealed that in the UC model group, there was a notable rise in the proportion of Bacteroidetes and a corresponding decrease in Firmicutes. However, following the BTWT intervention, this pattern was efficiently flipped. *Firmicutes* play an irreplaceable role in the maintenance of intestinal health through the production of beneficial metabolites (secondary bile acids, butyrate, and indole metabolites), which are mainly manifested in the enhancement of intestinal immunity and intestinal barrier function (Liu et al. 2021). In addition, in this study, the abundance of *Bifidobacterium*, *Turicibacter*, *Dubosiella* and *Alistipes* increased after BTWT intervention. *Bifidobacterium* can guide the differentiation of immature colonic macrophages through metabolic source of indole‐3‐lactic acid, and improve colitis‐associated tumorigenesis (Li et al. 2024). *Dubosiella newyorkensis* has become a potential target for the treatment of inflammatory bowel diseases by activating the AhR‐IDO1‐Kyn metabolic circuit in dendritic cells and mediating Treg‐mediated immunosuppressive microenvironment to regulate body immunity (Zhang, Tu, et al. 2024). *Alistipes onderdonkii* enhances the microbial metabolite 5HIAA, thereby activating the AhR signal and effectively improving DSS‐induced colitis (Zhong et al. 2024). Unlike the well‐established protective consensus for *Bifidobacterium*, *Turicibacter* exhibits marked context‐dependent functionality. *Turicibacter* plays a “double‐edged sword” role in colitis and has a dual role in protecting and promoting colitis. The protective effect of *Turicibacter* is often related to secondary bile acid metabolism (Lynch et al. 2023), whereas its pro‐inflammatory effects are typically associated Th17 responses (Fan et al. 2025). Limited by the inherent limitations of 16S rRNA sequencing in strain resolution, this study cannot determine whether the *Turicibacter* enriched by BTWT is a protective subtype or a pro‐inflammatory subtype. Therefore, we do not advocate that the efficacy of BTWT is simply attributed to the increased abundance of *Turicibacter*. On the contrary, we speculate that the overall anti‐colitis effect of BTWT is more likely to be due to the global regulation of the entire intestinal microecosystem and the synergistic change of the metabolite network, rather than the absolute number change of a single taxon.

Small molecules of tryptophan metabolism are intermediates that coordinate microbial‐host interactions and activate networks of related signals (e.g., AhR, GPRs, and PXR) that maintain host health. The findings from our study demonstrated a substantial rise in the amounts of tryptophan‐derived indole metabolites, for example: 3‐lactic acid (ILA), following the BTWT treatment. According to recent findings, supplementing with ILA seemed to shield mice from the ravages of DSS‐induced colitis. The underlying reason appears to be the suppression of glycolysis, NF‐κB, and HIF signaling pathways by ILA, which happens through the activation of AhR. This, in turn, resulted in a decrease in CCL2/7 production within the epithelium and helped tweak the interactions between epithelial and macrophage cells, thus helping to preserve the balance of the intestinal environment (Yu et al. 2023). Although we detected elevated ILA in both feces and serum following BTWT treatment, the local colonic mucosal ILA concentration, which is most relevant to epithelial AhR signaling, was not measured and warrants further investigation. In future studies, we also need to verify the ILA production capacity of specific candidate genera by in vitro culture or aseptic colonization experiments. Emerging research suggests that IAA could help alleviate the clinical manifestations of colitis by kickstarting the ERK signaling pathway, making it a promising avenue for potentially preventing or managing ulcerative colitis (Qu et al. 2024). Indole can inhibit Wnt signaling, inhibit intestinal tumor growth, and protect intestinal health (Wyatt and Greathouse 2021). At the same time, indole can also activate the Nrf2 signaling pathway and promote self‐repair of the intestinal mucosa, thereby reducing the damage caused by harmful substances to the intestine (Li et al. 2023). The results of our study indicate that BTWT effectively alleviates the clinical manifestations of colitis by modulating the microbial composition within the gut and influencing tryptophan metabolism, particularly through ILA and related metabolites, thereby restoring harmony to the intestinal microenvironment.

The aryl hydrocarbon receptor (AhR) can be found across diverse cellular populations, spanning immune, epithelial, endothelial, and neuronal cell types. Functioning as a transcription factor that requires ligand activation, AhR typically remains dormant in the cytoplasm until it encounters its binding partner, maintaining this inactive state through its association with molecular chaperones including HSP90, XAP2, and p23. Through the study of AhR gene modification or in vivo experiments with various AhR ligands, it is clear that AhR regulates multiple aspects of gastrointestinal function (Murray and Perdew 2017; Stockinger et al. 2014). When a ligand binds to it, the AhR protein moves into the nucleus, where it gets activated and kicks off the expression of CYP1A1 and CYP1B1 genes while also setting in motion the transcriptional control of various downstream genes (e.g., IL‐22, STAT3, etc.). AhR signaling can also inhibit the stemness program by transcriptionally regulating factors, such as RNF43, an E3 ubiquitin ligase. In addition, ligand‐activated AhR enhances intestinal epithelial cell junctions through increased expression of tight junction proteins (Stockinger et al. 2021). Although tryptophan indole metabolites are mostly natural ligands for AhR and can directly activate AhR, our results revealed that the protein and mRNA levels of *Ahr*, *Cyp1a1*, and *Cyp1b1* were differentially regulated after BTWT intervention, and the mRNA levels of *Il‐22*, *Il‐6*, and *Ifn‐γ* also showed different degrees of recovery. In addition, in vitro experiments on intestinal epithelial cells showed that within the safe concentration range of BTWT, BTWT did not affect the expression of AhR protein, nor did it affect the expression of its target proteins CYP1A1 and CYP1B1. Surprisingly, the tryptophan metabolite ILA significantly promoted AhR expression within the safe concentration range. Our results showed that BTWT could not directly activate AhR, while BTWT‐regulated metabolite ILA could activate AhR. In the future, we will further explore the activation of AhR, including nuclear translocation immunofluorescence or reporter gene detection. Whether BTWT can affect the AhR transcriptional regulation of RNF43 needs to be explored in future studies.

Research confirms that Chinese medicines' effectiveness hinges on their interaction with gut microbiota. On one hand, the gut microbiota can metabolize the active ingredients of Chinese medicines through hydrolysis and reduction to generate metabolites to improve the efficacy of UC; on the other hand, the active ingredients of Chinese medicines can directly contact the gut microbiota to promote beneficial bacteria, inhibit harmful bacteria, or regulate gut microbiota metabolites such as short‐chain fatty acids, bile acids, and tryptophan, which can play a therapeutic role in the treatment of UC (Zou et al. 2024). In our study, BTWT modulated the structure and diversity of the gut microbiota and regulated tryptophan metabolism in UC mice to exert its pharmacological effects, whereas the pharmacological effects were severely diminished in the antibiotic‐treated pseudo‐bacteriostatic mouse model. However, pathological damage was ameliorated in mice following FMT intervention. The partial restoration observed in the FMT group suggests that the gut microbiota contributes to, but does not fully account for, the therapeutic effects of BTWT. Consequently, our findings indicate that BTWT exerts a dual influence on gut microbiota‐modulating both its structural composition and diversity‐while simultaneously relying on these microbial communities to achieve its pharmacological benefits. These outcomes align closely with previously documented research in this field (Fan et al. 2020). Consequently, our findings indicate that BTWT treatment tipped the scales in favor of Firmicutes while putting the brakes on Bacteroidetes and Proteobacteria, leading to a notable uptick in tryptophan‐derived indole metabolites like ILA. This cascade of events, in turn, got the AhR signaling pathway humming and turned down the volume on inflammatory factor expression. This has a protective effect on experimental colitis, a finding that is similar to that reported by Dong et al. (2021). Based on the above, we hypothesize that BTWT, with its chemical composition, interacts with the gut microbiota while affecting tryptophan metabolism and improving the intestinal inflammatory state.

Although this study found a potential microbial‐metabolite‐host interaction axis that may be involved in mediating the therapeutic effect of BTWT on colitis, there are several limitations in this study. First, although our data show a strong correlation between BTWT treatment, AhR pathway upregulation and colitis improvement, the causal relationship remains to be determined. Future studies using AhR antagonists (such as CH‐223191) or siRNA will be of great significance to elucidate whether the protective effect of BTWT is mediated by AhR activation. Second, although our data consistently associate BTWT treatment with gut microbiota remodeling, elevated ILA, AhR pathway upregulation, and colitis amelioration, these findings are correlative rather than causal. We did not perform ILA‐specific loss‐of‐function experiments (e.g., genetic knockout of bacterial ILA synthesis pathways or ILA reconstitution in pseudo‐germ‐free mice) to definitively establish ILA as the primary active metabolite mediating BTWT's protective effects. Third, we acknowledge that the HT‐29/LTA model is a reductionist system that does not fully capture the complex immune‐epithelial interactions in UC. Our in vitro experiments were designed for targeted validation of ILA's direct epithelial effects based on in vivo metabolomics findings, and future studies incorporating immune cells or co‐culture systems are warranted to extend these observations. Fourth, antibiotic‐induced dysbiosis represents an inherent limitation of the microbial depletion model, and future studies using germ‐free mice are required to confirm these findings.

In summary, BTWT treatment was associated with gut microbiota–dependent reprogramming of tryptophan metabolism, increased ILA production, and AhR pathway activation, which correlated with the suppression of colonic inflammation. Critically, FMT partially recapitulated the therapeutic effects, suggesting that the gut microbiota contributes substantially to BTWT's efficacy. These findings establish that the microbiota‐ILA‐AhR axis may be the important mechanism by which BTWT alleviates colitis. Collectively, this work provides a robust mechanistic foundation for the clinical application of BTWT and opens new avenues for developing microbiota‐targeted or metabolite‐based therapeutics for UC.

## Author Contributions

**Chun Niu:** methodology, investigation, writing – review and editing, writing – original draft. **Yongli Hua:** funding acquisition, project administration, writing – review and editing. **Jianguo Kang:** methodology, data curation, validation. **Fang Li:** formal analysis, validation, supervision. **Baoxia Chen:** visualization. **Rongxia Guo:** resources, formal analysis. **Yanming Wei:** conceptualization.

## Funding

We would like to express our gratitude to the National Natural Science Foundation of China (32260898 and 31860717), the China Agriculture Research System of MOF and MARA (CARS‐36), the Fuxi Foundation of Gansu Agricultural University (Gaufx‐03J01), Science and Technology Program of Gansu Province (25JRRA385), and Gansu Provincial University Postgraduate “Innovation Star” (2025CXZX‐765) for supporting this study.

## Disclosure

All authors stated that this study was not helped by any AI or AI‐related technologies.

## Conflicts of Interest

The authors declare no conflicts of interest.

## Supporting information


**Figure S1:** Establishment of a microbial depletion mouse model using antibiotics. (A) Rate of change in body weight of mice. (B) Spleen weight. (C) Diagram of cecum anatomy. (D1‐4) Analysis of α‐diversity of the gut microbiota. (E1) PCA scatter plot. (E2) PCoA scatter plot. (E3) NMDS scatter plot. (F1) Changes in the gut microbial composition at the phylum level. (G1) Changes in the gut microbial composition at the genus level. (F2) Test for the significance of differences between groups at the phylum level. (G2) Test of the significance of differences between groups at the genus level. Data are expressed as the mean ± SEM, ***p < 0.001.

## Data Availability

Data related to this paper can be obtained from the corresponding authors upon request. The 16S rRNA raw data has been stored in Mendeley (https://data.mendeley.com/preview/f7m3kkr67d?a=e8f25e5f‐cb7b‐431f‐ac40‐741a80c84214).

## References

[fsn372434-bib-0001] Agus, A. , J. Planchais , and H. Sokol . 2018. “Gut Microbiota Regulation of Tryptophan Metabolism in Health and Disease.” Cell Host & Microbe 23: 716–724. 10.1016/j.chom.2018.05.003. 29902437

[fsn372434-bib-0002] Dong, S. , M. Zhu , K. Wang , et al. 2021. “Dihydromyricetin Improves DSS‐Induced Colitis in Mice via Modulation of Fecal‐Bacteria‐Related Bile Acid Metabolism.” Pharmacological Research 171: 105767. 10.1016/j.phrs.2021.105767. 34273490

[fsn372434-bib-0003] Fan, Q. , X. Guan , Y. Hou , et al. 2020. “Paeoniflorin Modulates Gut Microbial Production of Indole‐3‐Lactate and Epithelial Autophagy to Alleviate Colitis in Mice.” Phytomedicine 79: 153345. 10.1016/j.phymed.2020.153345. 33002829

[fsn372434-bib-0004] Fan, Y. Y. , Z. J. Yang , M. Y. Wang , et al. 2025. “Resveratrol Alleviates IBD‐Associated Neuropsychiatric Comorbidities via Microbiota‐Dependent Arginine Metabolism Reprogramming and Microglial M2 Polarization Through Gut‐Brain Axis.” Cell Communication and Signaling 23: 429. 10.1186/s12964-025-02448-w. PMC12512323 41074090

[fsn372434-bib-0005] Feuerstein, J. D. , A. C. Moss , and F. A. Farraye . 2019. “Ulcerative Colitis.” Mayo Clinic Proceedings 94: 1357–1373. 10.1016/j.mayocp.2019.01.018. 31272578

[fsn372434-bib-0006] Gao, X. , R. Sun , N. Jiao , et al. 2023. “Integrative Multi‐Omics Deciphers the Spatial Characteristics of Host‐Gut Microbiota Interactions in Crohn's Disease.” Cell Reports Medicine 4: 101050. 10.1016/j.xcrm.2023.101050. PMC10313922 37172588

[fsn372434-bib-0007] Gasaly, N. , P. de Vos , and M. A. Hermoso . 2021. “Impact of Bacterial Metabolites on Gut Barrier Function and Host Immunity: A Focus on Bacterial Metabolism and Its Relevance for Intestinal Inflammation.” Frontiers in Immunology 12: 658354. 10.3389/fimmu.2021.658354. PMC8187770 34122415

[fsn372434-bib-0008] Grossberg, L. B. , K. Papamichael , and A. S. Cheifetz . 2022. “Review Article: Emerging Drug Therapies in Inflammatory Bowel Disease.” Alimentary Pharmacology & Therapeutics 55: 789–804. 10.1111/apt.16785. 35166398

[fsn372434-bib-0009] Guo, Y. , Y. Li , Q. Cao , L. Ye , J. Wang , and M. Guo . 2022. “The Function of Natural Polysaccharides in the Treatment of Ulcerative Colitis.” Frontiers in Pharmacology 13: 927855. 10.3389/fphar.2022.927855. PMC9289104 35860025

[fsn372434-bib-0010] Hu, J. , H. Chen , L. Zhu , et al. 2025. “Baitouweng Decoction Modulates Gut Microbial Production of Indole‐3‐Propionic Acid and Epithelial Necroptosis to Alleviate DSS‐Induced Colitis in Mice.” Chinese Medicine 20: 119. 10.1186/s13020-025-01143-9. PMC12312286 40745657

[fsn372434-bib-0011] Kobayashi, T. , B. Siegmund , C. Le Berre , et al. 2020. “Ulcerative Colitis.” Nature Reviews. Disease Primers 6: 74. 10.1038/s41572-020-0205-x. 32913180

[fsn372434-bib-0012] Lavelle, A. , and H. Sokol . 2020. “Gut Microbiota‐Derived Metabolites as Key Actors in Inflammatory Bowel Disease.” Nature Reviews. Gastroenterology & Hepatology 17: 223–237. 10.1038/s41575-019-0258-z. 32076145

[fsn372434-bib-0013] Li, C. , J. Wang , R. Ma , et al. 2022. “Natural‐Derived Alkaloids Exhibit Great Potential in the Treatment of Ulcerative Colitis.” Pharmacological Research 175: 105972. 10.1016/j.phrs.2021.105972. 34758401

[fsn372434-bib-0014] Li, L. , P. Peng , N. Ding , W. Jia , C. Huang , and Y. Tang . 2023. “Oxidative Stress, Inflammation, Gut Dysbiosis: What Can Polyphenols Do in Inflammatory Bowel Disease?” Antioxidants 12: 967. 10.3390/antiox12040967. PMC10135842 37107341

[fsn372434-bib-0015] Li, Y. , Q. Li , R. Yuan , Y. Wang , C. Guo , and L. Wang . 2024. “ *Bifidobacterium breve* ‐Derived Indole‐3‐Lactic Acid Ameliorates Colitis‐Associated Tumorigenesis by Directing the Differentiation of Immature Colonic Macrophages.” Theranostics 14: 2719–2735. 10.7150/thno.92350. PMC11103503 38773969

[fsn372434-bib-0016] Li, Y. y. , X. j. Wang , Y. l. Su , et al. 2022. “Baicalein Ameliorates Ulcerative Colitis by Improving Intestinal Epithelial Barrier via AhR/IL‐22 Pathway in ILC3s.” Acta Pharmacologica Sinica 43: 1495–1507. 10.1038/s41401-021-00781-7. PMC9160000 34671110

[fsn372434-bib-0017] Liu, S. , W. Zhao , P. Lan , and X. Mou . 2021. “The Microbiome in Inflammatory Bowel Diseases: From Pathogenesis to Therapy.” Protein & Cell 12: 331–345. 10.1007/s13238-020-00745-3. PMC8106558 32601832

[fsn372434-bib-0018] Liu, X. , S. He , Q. Li , X. Mu , G. Hu , and H. Dong . 2020. “Comparison of the Gut Microbiota Between Pulsatilla Decoction and Levofloxacin Hydrochloride Therapy on *Escherichia coli* Infection.” Frontiers in Cellular and Infection Microbiology 10: 319. 10.3389/fcimb.2020.00319. PMC7344306 32714880

[fsn372434-bib-0019] Liu, Y. , B. G. Li , Y. H. Su , et al. 2022. “Potential Activity of Traditional Chinese Medicine Against Ulcerative Colitis: A Review.” Journal of Ethnopharmacology 289: 115084. 10.1016/j.jep.2022.115084. 35134488

[fsn372434-bib-0020] Lloyd‐Price, J. , C. Arze , A. N. Ananthakrishnan , et al. 2019. “Multi‐Omics of the Gut Microbial Ecosystem in Inflammatory Bowel Diseases.” Nature 569: 655–662. 10.1038/s41586-019-1237-9. PMC6650278 31142855

[fsn372434-bib-0021] Lynch, J. B. , E. L. Gonzalez , K. Choy , et al. 2023. “Gut Microbiota Turicibacter Strains Differentially Modify Bile Acids and Host Lipids.” Nature Communications 14: 3669. 10.1038/s41467-023-39403-7. PMC10281990 37339963

[fsn372434-bib-0022] Magro, F. , G. Cordeiro , A. M. Dias , and M. M. Estevinho . 2020. “Inflammatory Bowel Disease – Non‐Biological Treatment.” Pharmacological Research 160: 105075. 10.1016/j.phrs.2020.105075. 32653651

[fsn372434-bib-0023] Murray, I. A. , and G. H. Perdew . 2017. “Ligand Activation of the Ah Receptor Contributes to Gastrointestinal Homeostasis.” Current Opinion in Toxicology 2: 15–23. 10.1016/j.cotox.2017.01.003. PMC5604257 28944314

[fsn372434-bib-0024] Ni, J. , G. D. Wu , L. Albenberg , and V. T. Tomov . 2017. “Gut Microbiota and IBD: Causation or Correlation?” Nature Reviews Gastroenterology & Hepatology 14: 573–584. 10.1038/nrgastro.2017.88. PMC5880536 28743984

[fsn372434-bib-0025] Niu, C. , X. l. Hu , Z. w. Yuan , et al. 2023. “Pulsatilla Decoction Improves DSS‐Induced Colitis via Modulation of Fecal‐Bacteria‐Related Short‐Chain Fatty Acids and Intestinal Barrier Integrity.” Journal of Ethnopharmacology 300: 115741. 10.1016/j.jep.2022.115741. 36162543

[fsn372434-bib-0026] Niu, W. , X. Chen , R. Xu , et al. 2021. “Polysaccharides From Natural Resources Exhibit Great Potential in the Treatment of Ulcerative Colitis: A Review.” Carbohydrate Polymers 254: 117189. 10.1016/j.carbpol.2020.117189. 33357839

[fsn372434-bib-0027] Pan, S. M. , C. L. Wang , Z. F. Hu , et al. 2024. “Baitouweng Decoction Repairs the Intestinal Barrier in DSS‐Induced Colitis Mice via Regulation of AMPK/mTOR‐Mediated Autophagy.” Journal of Ethnopharmacology 318: 116888. 10.1016/j.jep.2023.116888. 37437793

[fsn372434-bib-0028] Qu, X. , Y. Song , Q. Li , et al. 2024. “Indole‐3‐Acetic Acid Ameliorates Dextran Sulfate Sodium‐Induced Colitis via the ERK Signaling Pathway.” Archives of Pharmacal Research 47: 288–299. 10.1007/s12272-024-01488-z. 38489148

[fsn372434-bib-0029] Reikvam, D. H. , A. Erofeev , A. Sandvik , et al. 2011. “Depletion of Murine Intestinal Microbiota: Effects on Gut Mucosa and Epithelial Gene Expression.” PLoS One 6: e17996. 10.1371/journal.pone.0017996. PMC3061881 21445311

[fsn372434-bib-0030] Rosen, M. J. , A. Dhawan , and S. A. Saeed . 2015. “Inflammatory Bowel Disease in Children and Adolescents.” JAMA Pediatrics 169: 1053–1060. 10.1001/jamapediatrics.2015.1982. PMC4702263 26414706

[fsn372434-bib-0031] Shen, Z. , Q. Zhou , Y. Ni , W. He , H. Shen , and L. Zhu . 2019. “Traditional Chinese Medicine for Mild‐To‐Moderate Ulcerative Colitis: Protocol for a Network Meta‐Analysis of Randomized Controlled Trials.” Medicine 98: e16881. 10.1097/MD.0000000000016881. PMC6831175 31415431

[fsn372434-bib-0032] Stockinger, B. , P. Di Meglio , M. Gialitakis , and J. H. Duarte . 2014. “The Aryl Hydrocarbon Receptor: Multitasking in the Immune System.” Annual Review of Immunology 32: 403–432. 10.1146/annurev-immunol-032713-120245. 24655296

[fsn372434-bib-0033] Stockinger, B. , K. Shah , and E. Wincent . 2021. “AHR in the Intestinal Microenvironment: Safeguarding Barrier Function.” Nature Reviews Gastroenterology & Hepatology 18: 559–570. 10.1038/s41575-021-00430-8. PMC7611426 33742166

[fsn372434-bib-0034] Sun, M. , N. Ma , T. He , L. J. Johnston , and X. Ma . 2020. “Tryptophan (Trp) Modulates Gut Homeostasis via Aryl Hydrocarbon Receptor (AhR).” Critical Reviews in Food Science and Nutrition 60: 1760–1768. 10.1080/10408398.2019.1598334. 30924357

[fsn372434-bib-0035] Ungaro, R. , S. Mehandru , P. B. Allen , L. Peyrin‐Biroulet , and J. F. Colombel . 2017. “Ulcerative Colitis.” Lancet 389: 1756–1770. 10.1016/S0140-6736(16)32126-2. PMC6487890 27914657

[fsn372434-bib-0036] Wyatt, M. , and K. L. Greathouse . 2021. “Targeting Dietary and Microbial Tryptophan‐Indole Metabolism as Therapeutic Approaches to Colon Cancer.” Nutrients 13: 1189. 10.3390/nu13041189. PMC8066279 33916690

[fsn372434-bib-0037] Xiao, Y. , Y. Q. Jia , W. J. Liu , et al. 2024. “Pulsatilla Decoction Alleviates DSS‐Induced UC by Activating FXR‐ASBT Pathways to Ameliorate Disordered Bile Acids Homeostasis.” Frontiers in Pharmacology 15: 1399829. 10.3389/fphar.2024.1399829. PMC11224520 38974033

[fsn372434-bib-0038] Yang, W. , and Y. Cong . 2021. “Gut Microbiota‐Derived Metabolites in the Regulation of Host Immune Responses and Immune‐Related Inflammatory Diseases.” Cellular & Molecular Immunology 18: 866–877. 10.1038/s41423-021-00661-4. PMC8115644 33707689

[fsn372434-bib-0039] Yoshida, M. , Y. Kinoshita , M. Watanabe , and K. Sugano . 2015. “JSGE Clinical Practice Guidelines 2014: Standards, Methods, and Process of Developing the Guidelines.” Journal of Gastroenterology 50: 4–10. 10.1007/s00535-014-1016-1. 25448314

[fsn372434-bib-0040] Yu, K. , Q. Li , X. Sun , et al. 2023. “Bacterial Indole‐3‐Lactic Acid Affects Epithelium–Macrophage Crosstalk to Regulate Intestinal Homeostasis.” Proceedings of the National Academy of Sciences of the United States of America 120: e2309032120. 10.1073/pnas.2309032120. PMC10636326 37903267

[fsn372434-bib-0041] Yu, L. C. H. 2018. “Microbiota Dysbiosis and Barrier Dysfunction in Inflammatory Bowel Disease and Colorectal Cancers: Exploring a Common Ground Hypothesis.” Journal of Biomedical Science 25: 79. 10.1186/s12929-018-0483-8. PMC6234774 30413188

[fsn372434-bib-0042] Zarrinpar, A. , A. Chaix , Z. Z. Xu , et al. 2018. “Antibiotic‐Induced Microbiome Depletion Alters Metabolic Homeostasis by Affecting Gut Signaling and Colonic Metabolism.” Nature Communications 9: 2872. 10.1038/s41467-018-05336-9. PMC6054678 30030441

[fsn372434-bib-0043] Zhang, J. , B. Lin , Y. Zhang , et al. 2024. “Baitouweng Decoction Alleviates Ulcerative Colitis by Regulating Tryptophan Metabolism Through DOPA Decarboxylase Promotion.” Frontiers in Pharmacology 15: 1423307. 10.3389/fphar.2024.1423307. PMC11224817 38974042

[fsn372434-bib-0044] Zhang, Y. , S. Tu , X. Ji , et al. 2024. “Dubosiella Newyorkensis Modulates Immune Tolerance in Colitis via the L‐Lysine‐Activated AhR‐IDO1‐Kyn Pathway.” Nature Communications 15: 1333. 10.1038/s41467-024-45636-x. PMC10864277 38351003

[fsn372434-bib-0045] Zhong, S. , Y. Q. Sun , J. X. Huo , et al. 2024. “The Gut Microbiota‐Aromatic Hydrocarbon Receptor (AhR) Axis Mediates the Anticolitic Effect of Polyphenol‐Rich Extracts From Sanghuangporus.” iMeta 3: e180. 10.1002/imt2.180. PMC11170970 38882491

[fsn372434-bib-0046] Zou, Y. , S. Wang , H. Zhang , et al. 2024. “The Triangular Relationship Between Traditional Chinese Medicines, Intestinal Flora, and Colorectal Cancer.” Medicinal Research Reviews 44: 539–567. 10.1002/med.21989. 37661373

