# Supplementary material for: Gut Microbiota‐Dependent Tryptophan Metabolism Drives the Therapeutic Effects of Baitouweng Tang in Colitis via the AhR Pathway
Source: Food Sci Nutr. 2026 Oct 14;14(10):e72434. doi: 10.1002/fsn3.72434 (PMC13662827; doi:10.1002/fsn3.72434)
Supplement: Supplementary file 1 [file FSN3-14-e72434-s001.docx]

**Gut Microbiota-Dependent Tryptophan Metabolism Drives the Therapeutic Effects of Baitouweng Tang in Colitis via the AhR Pathway**

Chun Niu^a^, Yongli Hua^a*^, Jianguo Kang^a^, Fang Li^b^, Baoxia Chen^a^, Rongxia Guo^a^, Yanming Wei^a^

^a^Institute of Traditional Chinese Veterinary Medicine, College of Veterinary Medicine, Gansu Agricultural University, Lanzhou 730070, China.

^b^Huating Animal Husbandry and Veterinary Bureau, Huating 744100, China.

^*^Corresponding author: Yongli Hua

Address: Gansu Agricultural University, No. 1 Yingmen Village, Anning District, Lanzhou, Gansu Province, 730070, China.

E-mail address: huayl@gsau.edu.cn, Tel: +86-931-13919082038.

Authors' E-mail addresses: [niuchun1999@163.com](mailto:niuchun1999@163.com) (Chun Niu); huayl@gsau.edu.cn (Yongli Hua); [2783329749@qq.com](mailto:2783329749@qq.com) (Jianguo Kang); [3486849282@qq.com](mailto:3486849282@qq.com) (Fang Li); [2669443762@qq.com](mailto:2669443762@qq.com) (Baoxia Chen); [15214043697@163.com](mailto:15214043697@163.com) (Rongxia Guo); [weiym@gsau.edu.cn](mailto:weiym@gsau.edu.cn) (Yanming Wei)

**Supplementary Figure S1.**

**A**

**B**

**C**

**D1**

**D2**

**D3**

**D4**

**E1**

**E2**

**E3**

**G2**

**G1**

**F2**

**F1**

**Supplementary Figure S1. Establishment of a microbial depletion mouse model using antibiotics.** (A) Rate of change in body weight of mice. (B) Spleen weight. (C) Diagram of cecum anatomy. (D1-4) Analysis of α-diversity of the gut microbiota. (E1) PCA scatter plot. (E2) PCoA scatter plot. (E3) NMDS scatter plot. (F1) Changes in the gut microbial composition at the phylum level. (G1) Changes in the gut microbial composition at the genus level. (F2) Test for the significance of differences between groups at the phylum level. (G2) Test of the significance of differences between groups at the genus level. Data are expressed as the mean ± SEM, ****P* < 0.001.
